# Novel chalcone-derived pyrazoles as potential therapeutic agents for the treatment of non-small cell lung cancer

**DOI:** 10.1038/s41598-022-07691-6

**Published:** 2022-03-08

**Authors:** Natalia Maciejewska, Mateusz Olszewski, Jakub Jurasz, Marcin Serocki, Maria Dzierzynska, Katarzyna Cekala, Ewa Wieczerzak, Maciej Baginski

**Affiliations:** 1 Faculty of Chemistry, Gdansk University of Technology, Gabriela Narutowicza 11/12, 80-233 Gdańsk, Poland; 2 Faculty of Chemistry, University of Gdansk, Wita Stwosza 63, 80-308 Gdańsk, Poland; 3 Ryvu Therapeutics, Leona Henryka Sternbacha 2, 30-394 Kraków, Poland

**Keywords:** Lung cancer, Microtubules, Apoptosis, Epithelial-mesenchymal transition

## Abstract

Lung cancer is considered to account for approximately one-fifth of all malignant tumor-related deaths worldwide and is therefore one of the most lethal malignancies. Pyrazole scaffold possesses a wide range of biological and pharmacological activities, which play important roles in medicinal chemistry. The present study reports the synthesis and in vitro biological characterization of nine pyrazoles derived from chalcones as potential anticancer agents for non-small cell lung cancer A-549, H226, and H460 cell lines. Most of the compounds efficiently inhibited the growth of all the tested cancer cell lines at micromolar concentrations. One of the most active compounds (**PCH-1**) was further evaluated for its effect on cell cycle distribution, apoptosis, migration, epithelial–mesenchymal transition, and oxidative stress. Furthermore, studies on the mechanism of action revealed that **PCH-1** disrupts microtubule assembly, leading to cancer cell death. Molecular modeling studies confirmed the potent interaction of **PCH-1** with the vinblastine binding site on tubulin. Overall, this study provides novel opportunities to identify anticancer agents in the pyrazole series.

## Introduction

Chalcones are an important class of natural compounds from the flavonoids family and are present in many edible plants such as fruits (tomatoes and citrus), nuts, vegetables, and tea^1^. Chalcone family members have often been used in traditional medicine and possess a large number of biological properties, including anticancer, antibacterial, antifungal, antimalarial, antidiabetic, anti-inflammatory, and antioxidant activities^2^. Several chalcones are currently being used in clinical practice or are undergoing clinical trials, exemplified by the selected analogs shown in Table 1. Chalcones have received considerable attention not only because of their broad spectrum of activities but also because of ease of synthesis and modification of the main core and good safety profile pertinent for oral administration^3^.

**Table 1:** Chemical structures of approved and clinically tested chalcones.

Chemical structure	Activity	Mechanism of action	References
	Choleretic Diuretic	PCSK9 inhibitor	^4^
	Anti-ulcer Mucoprotective	H2-receptor antagonist	^5^
	Antioxidant Anti-inflammatory	EC 1.14.18.1 inhibitor NMDA receptor antagonist GABA-A benzodiapine receptor positive allosteric modulator	^6^
	Anticancer Antioxidant Anti-inflammatory	Farnesoid X receptor agonist Inhibitor NFkB Inhibitor NRF2	^7,8^
	Vascular protective Antioxidant Anti-inflammatory Neuroprotective	Inhibitor oxidative stress Inhibitor cytokine production Inhibitor NF-κB	^9–11^

Chemically, chalcones have a scaffold of 1,3-diphenylprop-2-en-1-one either in *trans* or *cis* isomerism with two aromatic rings linked by a three-carbon α,β-unsaturated carbonyl system^12^. The predominant configuration is trans-isomer that is more stable thermodynamically^4^. Structural modification mainly focuses on both phenyl rings. Similar to naturally occurring chalcones, methoxy and hydroxy groups in positions on the phenyl rings are important for anticancer activities of chalcone derivatives^13^. Marquina et al. reported that 2′-hydroxy-4′-alkoxy chalcone derivatives exhibit strong cytotoxic activity against PC-3 cells by inducing a G2/M phase arrest and mitochondrial apoptotic pathway^14^. Wang et al. found that chalcones bearing the 3-hydroxyl-4-methoxy phenyl moiety demonstrated high cytotoxic activity against the MCF-7 breast cancer cell line and displayed potent tubulin polymerization inhibitory activity^15^. Moreover, Pawlak et al. established that 2′-hydroxy-2″,5″-dimethoxychalcone exerted a potent antitumor activity on canine lymphoma and leukemia cells and that the addition of two methoxy groups increased the proapoptotic activities of chalcones^16^.

Structurally, methoxylated chalcones are related to natural combretastatin A-4 and colchicine because of congruous spatial orientation between two aromatic rings^17^. Similar to combretastatin and colchicine, methoxylated chalcones bind to tubulin and prevent its polymerization, leading to cell cycle arrest by interruption of mitotic spindle assembly and resulting in cell death^18^. The methoxy substituent in A ring of chalcones appeared to be the crucial pharmacophoric group for the inhibition of tubulin polymerization^19,20^. Moreover, structural manipulations that vary in the basic structural framework or substitutions pattern have also been reported for antitumor properties, such as phenoxy moiety in the ortho position of the phenyl ring B^21^, methyl substitution in α position^8^, pyrazole hybrids^22^, and many others^23^.

Pyrazoles are heterocyclic compounds that possess two adjacent nitrogen atoms of the 5-membered ring of three carbon atoms. The pyrazole nucleus has diverse biological properties and therapeutic applications, such as analgesic, anticancer, anti-inflammatory, antimicrobial, antiviral, antidiabetic, anticonvulsant, antipyretic, and antidepressant properties^24,25^. Because many studies have reported the importance of the heterocyclic moiety of the pyrazole ring in medicinal chemistry, skeletons of pyrazoles are often modified to ameliorate their biological activity. Among others, pyrazoles derived from chalcones display an increase in the potency and spectrum of activity^26^.

On the basis of discussed studies and considering a constant need to develop new potent and selective anticancer compounds, we have designed and synthesized a series of derivatives of chalcone-derived pyrazoles with various combinations of substituents in the phenyl rings. Novel compounds were evaluated for their antitumor activity against various lung cancer cell lines, including studies on cytotoxic, antiproliferative, prooxidant, and proapoptotic effects. Among these compounds, **PCH-1** exhibited the strongest cytotoxic activity through interaction with tubulin, leading to cell cycle arrest and induction of apoptosis. To determine the molecular mechanism of action of these compounds, we performed molecular modeling studies, namely molecular docking. Our studies confirmed the potent interaction of **PCH-1** with the vinblastine binding site on tubulin. Overall, we have demonstrated that **PCH-1** has a potent antitumor potential for the treatment of lung cancer and could be a lead for new drug development.

## Results

### Design of compounds

**Figure 1:**
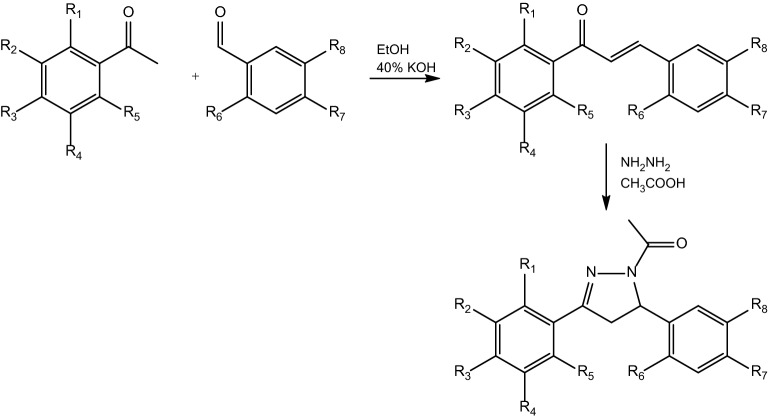
General synthetic pathway of the chalcone-derived pyrazoles.

The chalcone-derived pyrazoles were synthesized according to the protocols described by Yin Luo et al.^27^ and are presented in Table 2 (Fig. 1).

**Table 2:** Synthesized new chalcone-derived pyrazoles.

Compound	R_1_	R_2_	R_3_	R_4_	R_5_	R_6_	R_7_	R_8_
PCH-1	H	O-CH_2_-O		H	OCH_3_	H	OCH_3_	H
PCH-2	H	OCH_3_	OCH_3_	OCH_3_	H	H	O-CH_2_-O	
PCH-3	H	H	CH_3_	H	H	H	O-CH_2_-O	
PCH-4	OCH_3_	H	H	H	H	H	O-CH_2_-O	
PCH-5	H	O-CH_2_-O		H	H	H	O-CH_2_-O	
PCH-6	H	H	OCF_3_	H	H	H	O-CH_2_-O	
PCH-7	H	H	OCH_3_	H	H	H	CH_2_-CH_2_-O	
PCH-8	H	H	OCH_3_	H	H	OCH_3_	O-CH_2_-O	
PCH-9	H	S-CH_2_-O		H	OCH_3_	H	OH	H

Compounds PCH contain two differently substituted aromatic rings and *N*-acylated pyrazole ring. PCH-1, PCH-2, PCH-3, PCH-4, PCH-5, PCH-6, PCH-8 have at least one 1,3-benzodioxole moiety which is described as the structural element of compounds exhibiting an anticancer potential^28^. In PCH-7 and PCH-9 the oxygen atom in the benzodioxole ring is bioisosterically replaced by –CH_2_– group and sulfur atom, respectively. PCH-1, PCH-2, PCH-4, PCH-7, PCH-8, and PCH-9 have from one to three methoxy groups substituting aromatic rings. In case of PCH-3 the methoxy group is changed into methyl group. Fluorine atoms have been incorporated into the methoxy group in PCH-6 derivative.

### PCH-1 compound shows the potent cytotoxic effect towards NSCLC and non-toxic to normal cells

All the synthesized chalcone-derived pyrazoles were evaluated for their antiproliferative activity against three human non-small cell lung cancer cell lines: A-549, H226, and H460, and a nonmalignant human embryonic kidney cell line HEK293. Cisplatin and 5-fluorouracil (5-FU) were used as a reference. Cells were exposed to a range of PCH concentrations (0.5–100 µM) for 72 h and analyzed by 3-(4,5-dimethylthiazol-2-yl)-2,5-diphenyl-2H-tetrazolium bromide (MTT) assay. The results of the studies are expressed as the IC_50_ concentration that inhibits 50% growth of cells. As depicted in Table 3, **PCH-1** was found to be the most potent cytotoxic compound against A-549 cells among all the tested pyrazoles, with the IC_50_ value of 4.32 ± 0.28 µM. H226 and H460 cells showed similar sensitivity to **PCH-1** treatment, with IC_50_ values of 4.69 ± 0.43 µM and 8.40 ± 1.10 µM, respectively. Other compounds exhibited moderate activity against cancer cells, besides **PCH-2**, **PCH-3**, **PCH-4**, **PCH-7**, and **PCH-8**, which were active only against H226 and H460 cells. Following treatment with **PCH-1,** the viability of all the tested cancer cell lines decreased in a concentration-dependent manner. In contrast, the nonmalignant human embryonic kidney cells (HEK293) exhibited less reduction of cell viability after **PCH-1** treatment. This finding indicates that compound **PCH-1** inhibited the cell viability of cancer cells without significantly affecting normal kidney cells. Additionally, **PCH-1** showed superior activity to the reference compound Cisplatin by approximately 6.7-fold, 3.7-fold, and 2.6-fold more for A-549, H226, and H460 cells, respectively.

**Table 3:** In vitro growth inhibitory activity of pyrazoles and reference compounds presented as an IC_50_ ± SD (µM) value representing a concentration that inhibits 50% of cell growth.

Compound	A-549	H226	H460	HEK293
PCH-1	4.32 ± 0.28	4.69 ± 0.43	8.40 ± 1.10	20.95 ± 2.34
PCH-2	> 100	40.46 ± 3.52	43.84 ± 1.12	> 100
PCH-3	> 100	86.70 ± 0.92	24.53 ± 1.74	> 100
PCH-4	> 100	86.70 ± 4.70	63.43 ± 3.34	> 100
PCH-5	60.89 ± 4.30	57.99 ± 2.56	38.84 ± 1.77	46.99 ± 3.18
PCH-6	21.46 ± 4.26	24.36 ± 2.13	18.12 ± 2.13	42.90 ± 1.23
PCH-7	> 100	41.49 ± 1.12	30.16 ± 2.14	> 100
PCH-8	> 100	61.11 ± 3.12	30.16 ± 2.14	> 100
PCH-9	79.46 ± 4.97	38.38 ± 3.53	43.77 ± 3.97	34.43 ± 2.01
Cisplatin	29.01 ± 0.12	17.47 ± 2.12	21.49 ± 1.87	28.45 ± 1.97
5-FU	8.12 ± 1.01	4.01 ± 0.76	4.08 ± 0.43	11.09 ± 1.53
Etoposide	0.54 ± 0.21	1.03 ± 0.16	0.05 ± 0.01	1.91 ± 0.97

**Figure 2:**
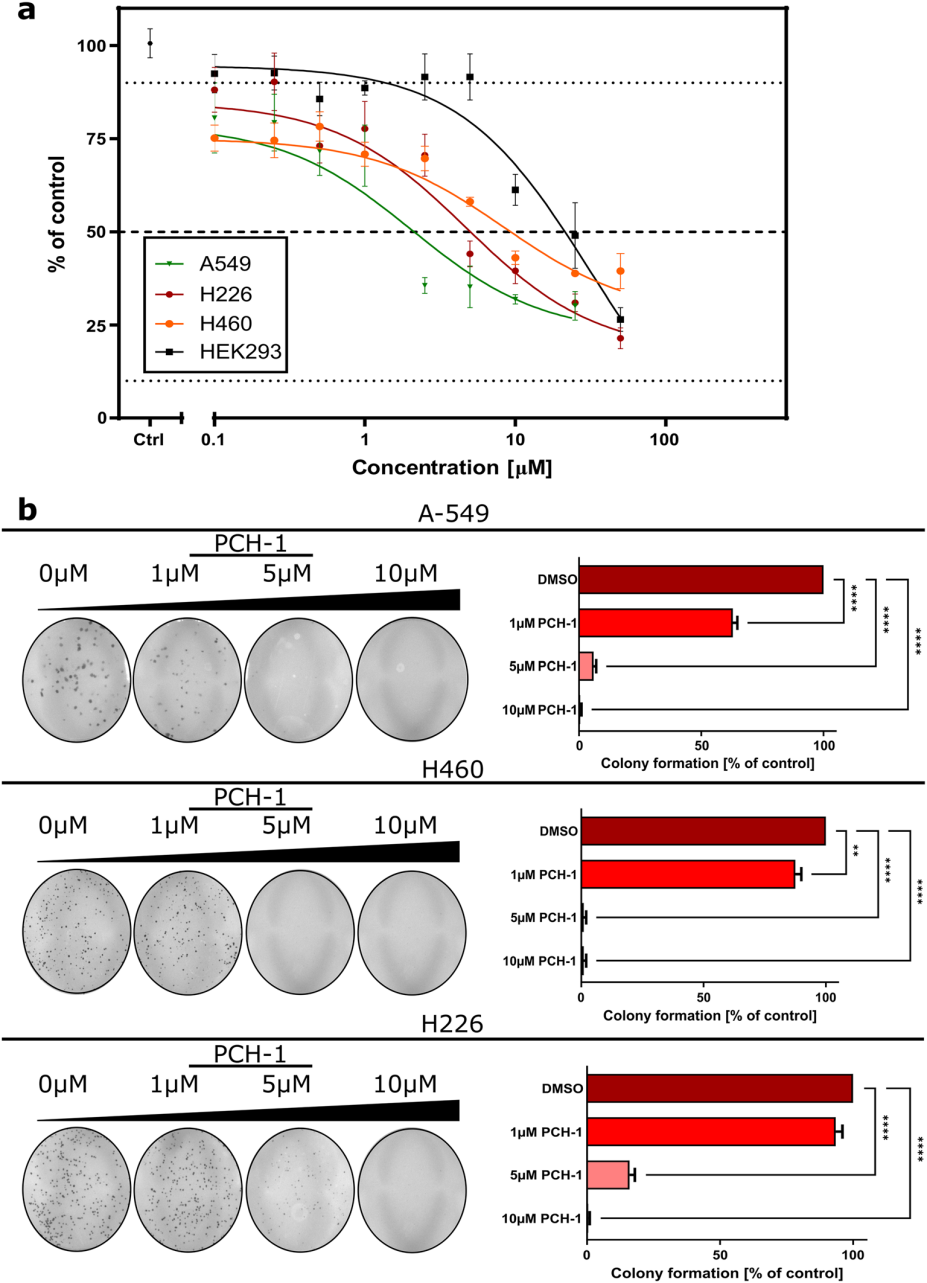
(**a**) Effect of compound **PCH-1** on cell viability after 72 h incubation with compound. Results of percent viability vs concentration were plotted, and the IC_50_ ± SEM was calculated for each cell line in triplicates by using GraphPad prism 9; (**b**) Representative photos of the colony formation assay are presented on the left panel and its quantification is depicted on the right panel. Data represent the mean ± SEM of three independent experiments. Statistical differences were analyzed by one-way ANOVA and post hoc Dunnett`s test. ** p < 0.001, **** p < 0.00001.

The most potent compound **PCH-1** was selected to test its antiproliferative property by using a colony formation assay. As depicted in Fig. 2, **PCH-1** treatment reduced the number of colonies in all the tested cell lines in a dose-dependent manner.

### PCH-1 arrest cells in G2/M checkpoint

**Figure 3:**
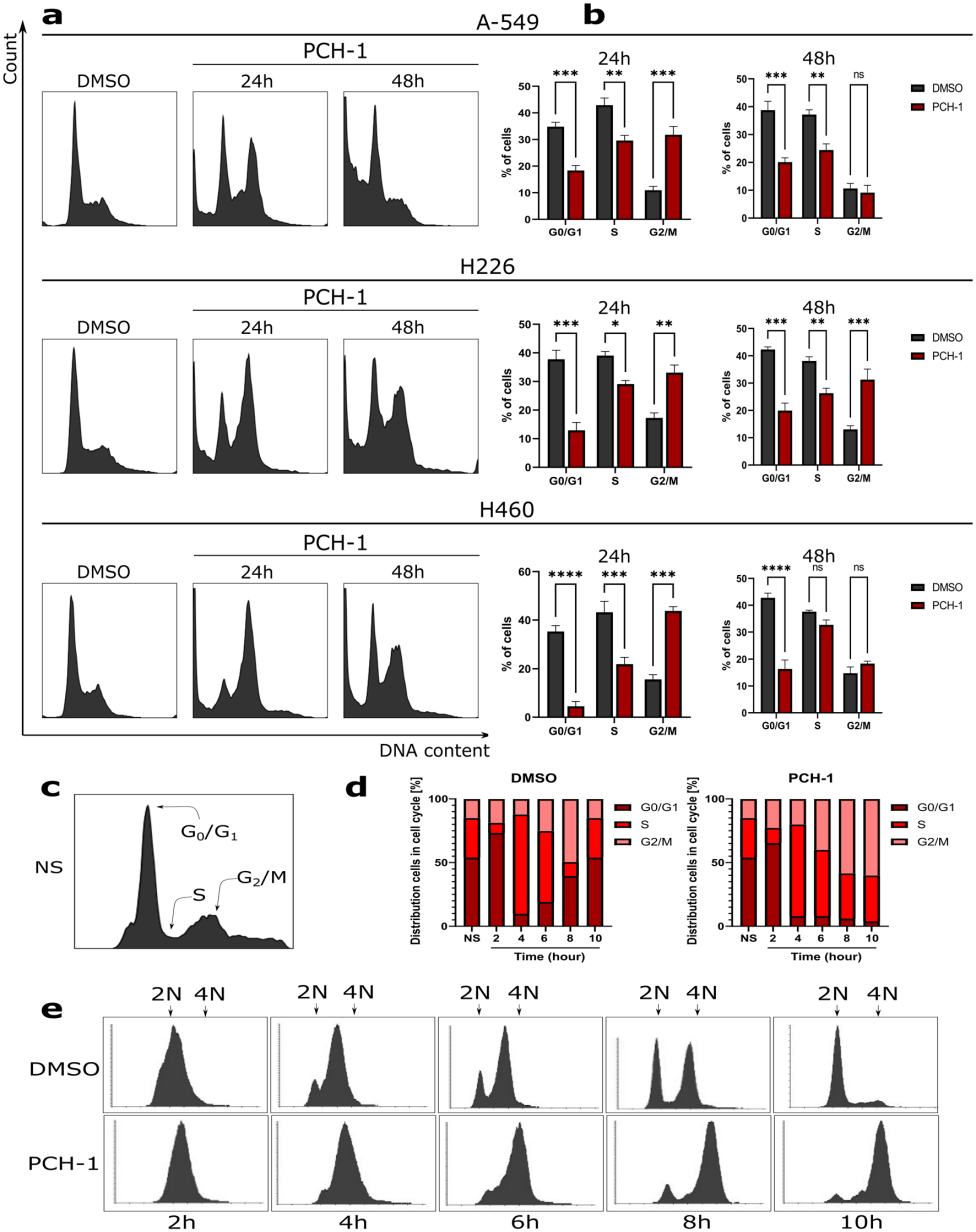
Cell cycle analysis of A-549, H226, and H460 cells after **PCH-1** treatment. (**a**) Representative histograms after DNA staining; (**b**) Statistical analyses of histograms. Error bars represent the SEM of data obtained in n = 3 independent experiments. Statistical differences were analyzed with one-way ANOVA and post hoc Dunnet’s test. *p < 0.01, **p < 0.001, ***p < 0.0001, ****p < 00001 vs. vehicle; (**c**) Determination of cycle phases in unsynchronized cells; (**d**) The quantitation of the flow cytometric analysis in A-549 synchronized cells; (**e**) Representative histograms after flow cytometric analyses of cells synchronized in the G_1_ phase after DMSO and **PCH-1** treatment. 2 N and 4 N represent the DNA content of a cell in the G1 (diploid), and G2 phase (tetraploid), respectively.

Promising antiproliferative properties of **PCH-1** led us to further investigate the effect of this compound on cell cycle distribution. Therefore, cell cycle progression was analyzed by quantifying DNA content using flow cytometry. As shown in Fig. 3a,b, treatment of A-549, H226, and H460 cells with **PCH-1** after 24 h significantly (**p < 0.001) induced cell cycle arrest in the G_2_/M phase, and this effect was followed by a decreasing trend at 48 h as compared to control. Concomitantly, we observed a significant increase in the sub-G_1_ population of cells with the highest peak at 48 h after **PCH-1** treatment, thus suggesting apoptotic cell death. To thoroughly analyze the course of the cycle, A-549 cells were synchronized with a double-thymidine block in the G_1_ phase and treated with IC_90_ concentration of **PCH-1** for the indicated time. DNA content was quantified by the flow cytometric method and presented in Fig. 3c–e. Slight alterations of the division cycle were visible after 2 h of incubation with the compound. The prolonged exposure to **PCH-1** resulted in the accumulation of cells at the G_2_/M phase, resulting in a 50.12% increase in population as compared to that of the control. After 10 h of thymidine release, the DMSO control group showed a typical pattern of the cell cycle in the G_1_/G_0_, S, and G_2_/M phases. The results indicated that **PCH-1** arrests cells in the G_2_/M checkpoint and halts cell mitosis in a time-dependent manner, leading to the inhibition of the growth of A-549 cells.

### PCH-1 disrupt microtubule assembly

**Figure 4:**
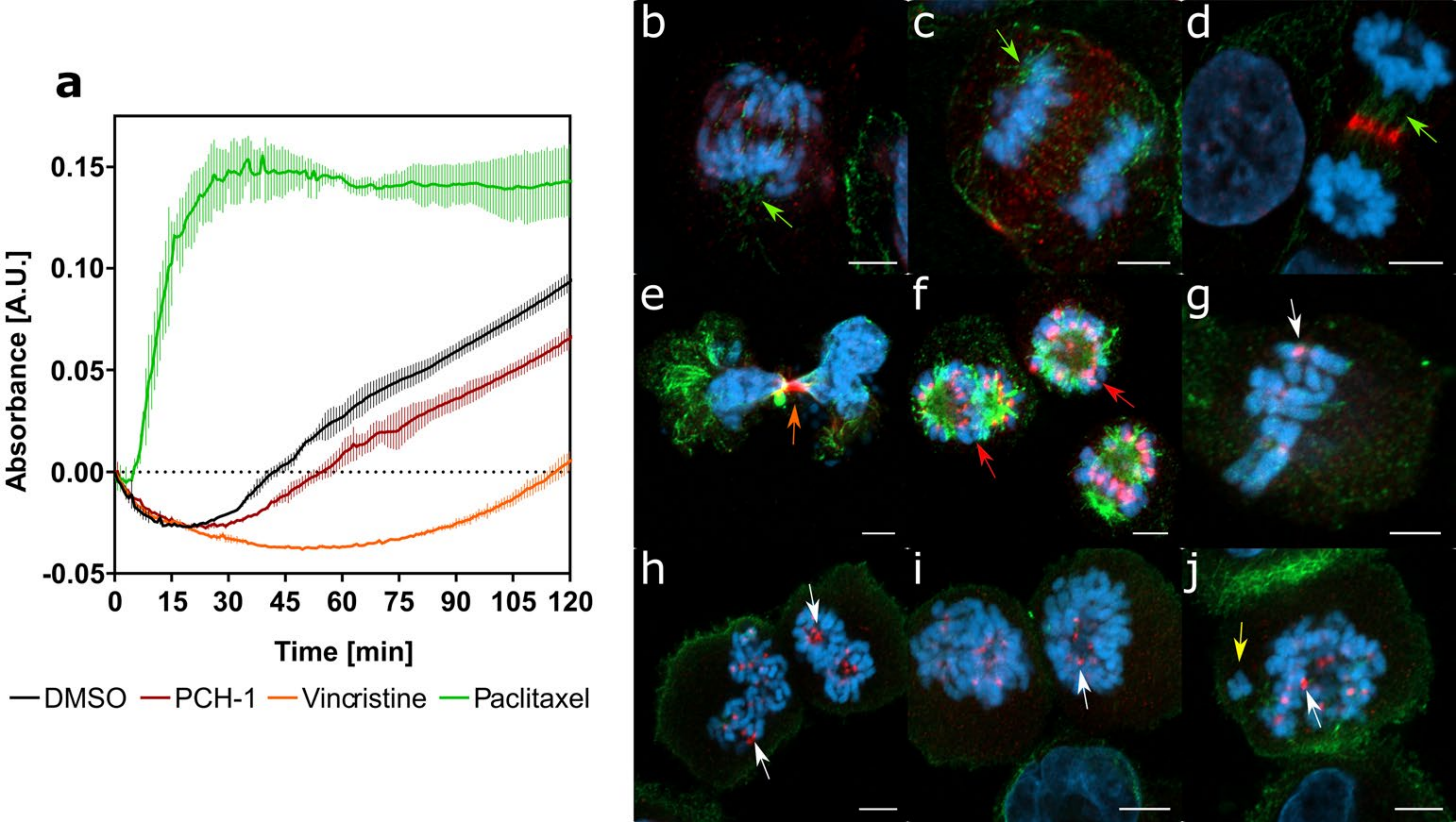
(**a**) Kinetic curves of a 120-min kinetic reaction of tubulin polymerization. Data represent the mean ± SEM of two independent experiments. Representative microscopy images (**b–j**) presenting immunofluorescence of microtubule assembly and Aurora B in A-549 cells after 6 h of treatment with **PCH-1**. Scale bars = 5 µM. DMSO, Vincristine, and Paclitaxel were used as reference compounds. The microtubule is depicted in green, Aurora B in red, and the nucleus is stained with DAPI (blue). (**b–d**) Mitosis in DMSO-treated cells; (**e**–**f**) Mitosis in Paclitaxel-treated cells; (**g**) Mitosis in Vincristine-treated cells; (**h–j**) Mitosis in **PCH-1**-treated cells. The arrows indicate a proper spindle assembly (green), a strong centromeric localization of Aurora B (white), chromosome scattering (yellow), improperly formed midbody (orange), multipolar spindle (red).

To further evaluate whether the tubulin–microtubule system was the target site of **PCH-1**, in vitro microtubule polymerization assays were performed using the microtubule protein with Paclitaxel and Vincristine as a reference. Paclitaxel and Vincristine are antimitotic drugs that have been commonly used in conventional chemotherapy for decades^29^. In 1979, it was shown that Paclitaxel accelerates the initiation of tubulin fiber polymerization and stabilizes the formed tubulin fibers in vitro, even in the absence of GTP or MAP in the solution^30^. Vincristine, on the other hand, binds to tubulin and inhibits its polymerization^31^. As shown in Fig. 4a, **PCH-1** remarkably reduced the total mass of tubulin polymer, without a significant increase in nucleation time. As expected, Paclitaxel accelerated the nucleation phase and increased the total mass of tubulin polymer, in contrast to Vincristine which extended the nucleation time and reduced the total mass of tubulin polymer compared to the vehicle. These results indicated that compound **PCH-1** interferes with microtubule polymerization by blocking microtubule assembly.

To confirm the influence of **PCH-1** on the organization of the tubulin cytoskeleton in A-549 cells, immunofluorescence was performed using antibodies for detecting β-tubulin and Aurora B. Aurora B is a protein kinase involved in the condensation and cohesion of chromosomes as well as in the process of attachment of tubulin spindles to the kinetochore and passing through the checkpoint of spindle assembly^32^. The expression of Aurora B is cell cycle-dependent and reaches a maximum at the G_2_/M transition, with the highest kinase activity during mitosis. Specifically, Aurora B localizes at the prophase in chromosomes, in the prometaphase and metaphase at centromeres, and is then transferred to the central mitotic spindle in the anaphase^33,34^. Visualization of the intracellular localization of Aurora B enables to determine whether the tubulin fibers forming the spindle are properly attached to the kinetochores and whether the cytokinesis process is proceeding properly.

A-549 cells after 6 h of exposure to the compounds showed a significantly increased fraction of mitotic cells. In the control group, usually 1 or 2 cells were seen dividing in several fields of view, while in the Paclitaxel, Vincristine, and **PCH-1** treatment groups, several mitotic cells could be seen in one field of view. As shown in Fig. 4b–d, mitosis is normal in control cells. A-549 cells treated with all compounds showed induction of various mitotic alterations that occurred mainly at the metaphase and anaphase where they induced a strong centromeric localization of Aurora B. Multipolar mitotic cells and scattered metaphases were visible in the Paclitaxel-treated group and Vincristine-treated group, respectively (Fig. 4e–g). Chromosome scattering with irregular distribution was observed in the equatorial plane, which implied either the absence or irregular arrangement of the spindle. The morphological changes in spindle structure after **PCH-1** treatment were nearly identical to those that occurred after Vincristine treatment and were the result of a prolonged metaphase arrest characterized by uncoordinated loss of chromatid cohesion (Fig. 4h–j).

**Figure 5:**
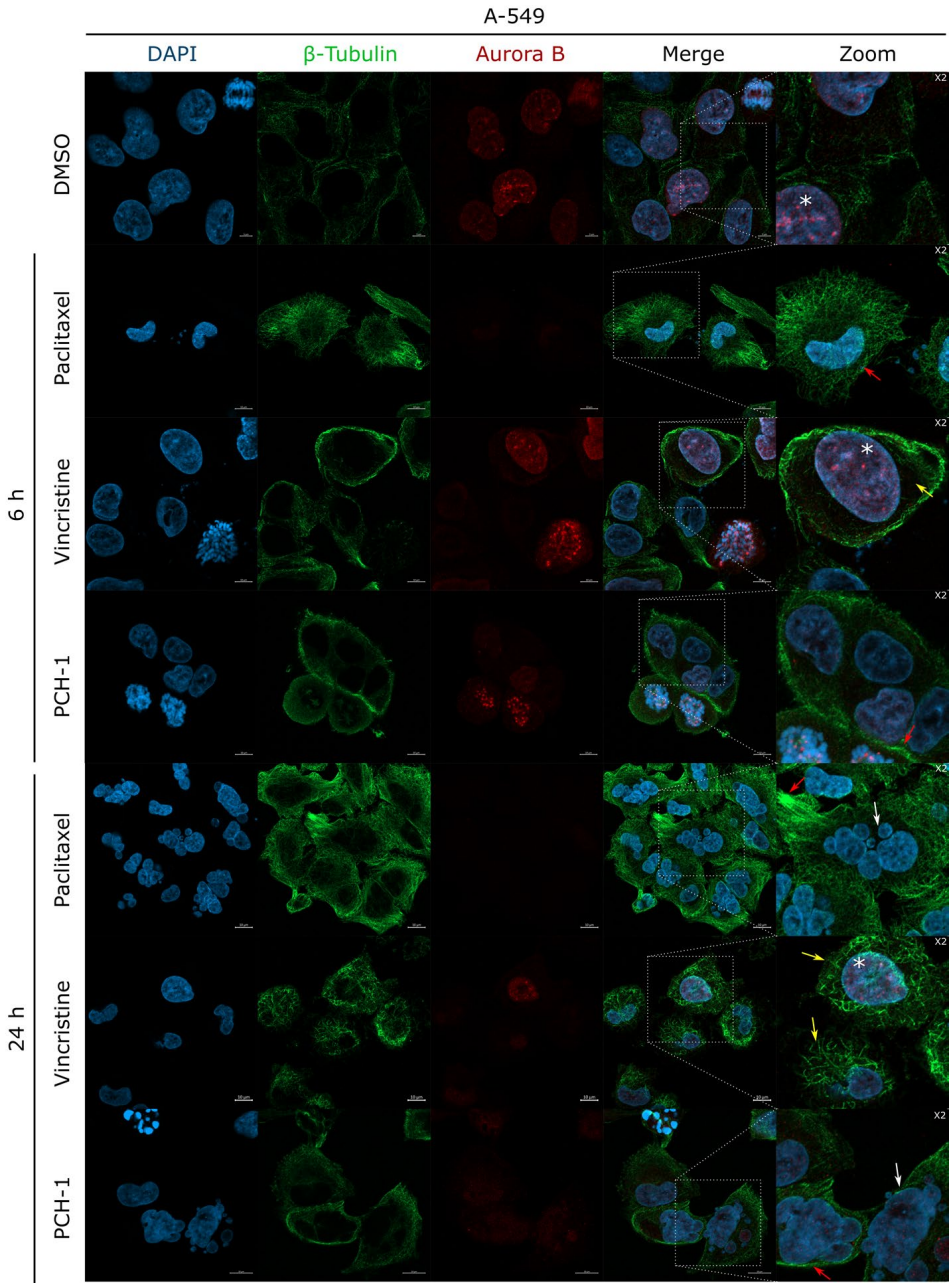
Representative microscopy images presenting immunofluorescence of microtubule assembly and Aurora B in A-549 cells at 6, and 24 h of treatment with **PCH-1**. The microtubule is depicted in green, Aurora B in red, and the nucleus is stained with DAPI (blue). Scale bars = 10 μm. The arrows indicate a multinucleated nucleus (white), disturbed residual tubulin skeleton (yellow), coiled spiral aggregates of tubulin formation of rigid microtubule bundles (red). Asterisk marks cell nuclei in the G2 phase characterized by high Aurora expression.

As shown in Fig. 5, A-549 cells after treatment with Paclitaxel passed the G_2_/M checkpoint and entered mitosis, but its course was disturbed by the formation of a multipolar spindle. The strong accumulation of Aurora B on centrosomes indicates the incorrect attachment of microtubule fibers to the kinetochore and syntelic/monotelic chromosome orientation, which activates the spindle assembly checkpoint and mitotic arrest. Long-term exposure of cells to Paclitaxel leads to mitotic catastrophe, resulting in the formation of multinucleated polyploid cells. A similar cellular effect was observed after treatment of the cells with Vincristine and **PCH-1**. Figure 5 shows that A-549 cells exposed to Vincristine or **PCH-1** passed the G_2_/M checkpoint and entered mitosis; however, inhibition of tubulin polymerization blocked mitotic spindle formation. Potent accumulation of Aurora B on centromeres is caused by the lack of attachment of tubulin fibers to the kinetochore, which activates the checkpoint of the formation of the division spindle and leads to the arrest of cell proliferation in the mitotic phase. The consequence of this is a mitotic catastrophe and the formation of multinucleated polyploid cells.

### PCH-1 induce caspase-dependent apoptotic cell death

To examine whether the effect of **PCH-1** on reducing the viability of lung cancer cells was the result of inducing apoptotic cell death, A-549, H226, and H460 cells were treated with the compound for 6, 24, and 48 h, and analyzed by Annexin V/propidium iodide (PI) double staining using flow cytometry (Fig. S1 in supplementary information). After 6 h of incubation with **PCH-1**, the percent of live cells in all the tested cell lines significantly decreased and the fraction of Annexin V-positive cells concomitantly increased, thus confirming phosphatidylserine externalization and the ongoing process of apoptosis. At 24 h of treatment with the compound, a slight increase in both early (Annexin V(+)/PI(−)) and late (Annexin V(+)/PI(+)) apoptotic fractions was observed. Later exposure (48 h) to **PCH-1** demonstrated a potent increase in the late apoptotic phase, from approximately 5% to 30–60%, depending on the cell line for vehicle and **PCH-1**-treated cells, respectively. Simultaneously, under the same conditions, a constant number of Annexin V(−)/PI(+) cells was observed for all cell lines. PI can enter only those cells with damaged plasma membrane, which enables to differentiate early apoptotic cells from late apoptotic and necrotic cells. Overall, biochemical changes involved in apoptotic cell death were prevailing, whereas necrotic changes were not observed. Furthermore, typical phenotypic features of cells undergoing apoptosis were visualized by fluorescent microscopy in Supplementary Fig. S2 and described in Supplementary Information.

To explain the type of cell death that cancer cells undergo after treatment with **PCH-1**, the ability of the compound to activate caspases 3–7 was determined by flow cytometry. As depicted in Supplementary Fig. S3 in Supplementary Information, after 6 h of treatment, **PCH-1** activates executioner caspase-3/7 that executes apoptosis, as revealed by a 2.6-fold, 2.9-fold, and 3.15-fold increase in apoptosis for A-549, H226, and H460 cells, respectively, as compared to control. Later exposure to **PCH-1** resulted in a massive increase in subpopulation cells with activated caspase 3/7, sustaining at a level greater than 5.8 times in A-549 cells and 4.7 times in H226 cells than in the control, while remaining approximately constant in H460 cells. The most pronounced activation of caspase 3 occurred equally in A-549 and H226 cell lines after 48 h of treatment and was sevenfold greater than that in the vehicle.

### PCH-1 deregulates several apoptotic and EMT-related proteins

**Figure 6:**
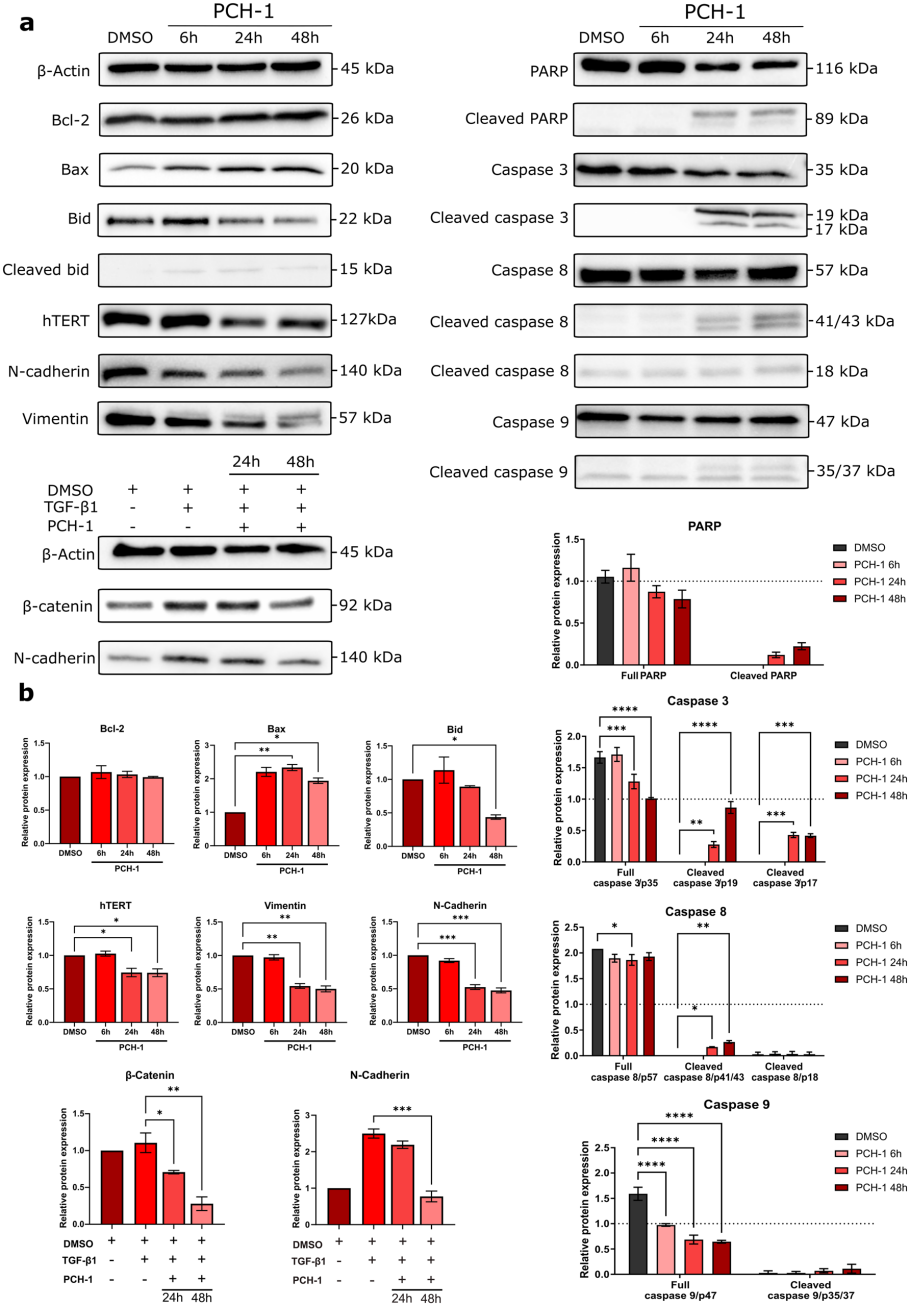
(**a**) Western blotting analysis showing the effects of **PCH-1** on the expression of apoptotic, and EMT-related proteins in the A-549 cell line. Full length western blots were displayed in Supplementary Fig. S4 in Supplementary Information; (**b**) Density ratios of protein expression level after treatment with **PCH-1** relative to β-Actin as a loading control. Data represent mean ± SEM of three independent experiments. Statistical differences were analyzed by one-way ANOVA and post hoc Dunnett’s test. * p < 0.01, ** p < 0.001, *** p < 0.0001, **** p < 0.00001.

To investigate the potential molecular mechanisms responsible for **PCH-1-**induced apoptosis in A-549 cells, we measured the expression of apoptosis-related proteins by western blot analysis. As shown in Fig. 6, **PCH-1** treatment downregulated the expression of full-length caspase 3, caspase 8, and caspase 9, accompanied by an increase in the levels of their cleaved forms. The activation of the caspase cascade by the test compound leads to proteolytic cleavage of poly(ADP-ribose) polymerase-1 (PARP-1), as revealed by a decrease in its full-length expression. Next, we determined the role of the antiapoptotic protein Bcl-2 as well as the proapoptotic proteins Bax and Bid in **PCH-1-**induced cell death in A-549 cells. As shown in Fig. 6, compared to the control cells, **PCH-1** treatment led to a significant increase in the Bax protein level (6 h, 24 h: **p < 0.001; 48 h: *p < 0.01), whereas the level of Bcl-2 was not changed. Additionally, we observed Bid cleavage (48 h: *p < 0.01), which was noted as a reduction in full-length Bid protein.

Epithelial-mesenchymal transition (EMT) plays a crucial role in tumorigenesis by promoting cell motility, migration, and metastasis. Thus, we examined the expression of proteins involved in the EMT process by western blot assays. Figure 6 shows that compound **PCH-1** dysregulated both biomarkers *N*-cadherin and vimentin of mesenchymal cells. The activity of *N*-cadherin in A-549 cells was reduced by 45% at 24 h (*p* = 0.0002) and by 50% at 48 h (*p* = 0.0002), while vimentin activity was reduced by 43% at 24 h (*p* = 0.005) and by 46% at 48 h (*p* = 0.004). Additional **PCH-1** inhibited Transforming Growth Factor β1 (TGF-β1) induced mesenchymal-like phenotype in A-549 cells, indicating downstream of *N*-cadherin by 66% at 48 h (p = 0.0003), and another EMT effector β-catenin, which was suppressed by 31% at 24 h (p = 0.0275) and by 66% at 48 h (p = 0.0019). Moreover, 24 and 48 h exposure to **PCH-1** significantly decreased (*p* = 0.001) the expression of the catalytic subunit of the telomerase reverse transcriptase hTERT, which is not only responsible for EMT but also plays an essential role in the maintenance of chromosomal telomere length, stemness, and resistance.

**Figure 7:**
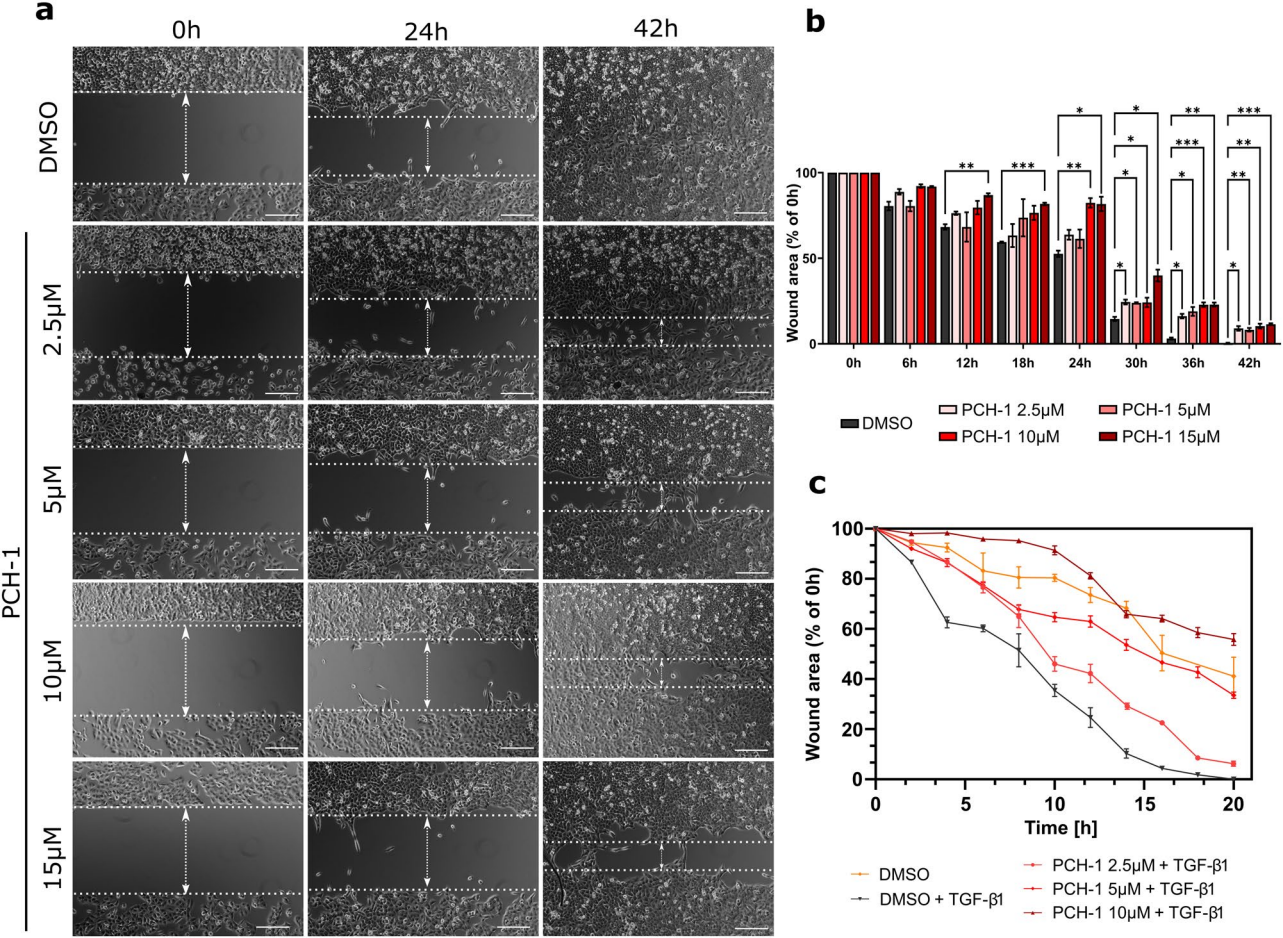
Analysis of cell migration by in vitro wound healing assay. (**a**) Time-lapse microscopy images after culture insert removal. Scale bars = 100 μm; (**b**) Quantification of the wound area after treatment A-549 cells with increasing concertation’s of **PCH-1**. Data represent mean ± SEM of three independent experiments. Statistical differences were analyzed by one-way ANOVA and post hoc Dunnett’s test. ** *p* < 0.001, *** *p* < 0.0001, **** *p* < 0.00001; (**c**) Quantification of wound area after treatment TGF-β1 stimulated A-549 cells with increasing concentrations of **PCH-1**.

To further confirm the antimigratory potential of **PCH-1**, the wound healing assay was performed on A-549 cells. As shown in Fig. 7, cells in the DMSO control group gradually repopulated the wound area in a time-dependent manner. A similar effect was observed in groups treated with 2.5 µM and 5 µM **PCH-1**, except for 30–36 h, where the ability of cells to repopulate the wounded area was temporarily attenuated as compared to that in the vehicle. In contrast, 10 and 15 µM concentrations of **PCH-1** significantly reduced the migration of the cells. This effect was retained during the entire exposure time, with the highest difference during 18–24 h compared to that in the control group. Finally, the anti-migratory properties of **PCH-1** were studied by co-treatment A-549 cells with TGF-β1. As shown in Fig. 7c, and Supplementary Fig. S5 in Supplementary Information, TGF-β1 alone accelerated wound closure, whereas **PCH-1** inhibited TGF-β1—induced cell motility, and as a result, the wound remained opened after 20 h of treatment with 5 µM and 10 µM of **PCH-1**. 2.5 µM of **PCH-1** also resulted in a decrease in the rate of cell migration, but after 20 h of exposure, the wound closed similarly to the control-treated with TGF-β1 alone. A noteworthy aspect is that we did not observe detached cells at any point of treatment. Moreover, studied concentrations of **PCH-1** were only slightly cytotoxic as evidenced by the IC50 values determined after 24 and 48 h of treatments, which were 41.27 ± 0.75 µM, and 25.58 ± 0.97 µM, respectively. This suggests that the observed antimigratory properties are not affected by the cytotoxic effect of the tested compound.

### PCH-1 induce intracellular oxidative stress

The generation of intracellular reactive oxygen species (ROS) causes an imbalance of cellular redox homeostasis, followed by damage of cellular components and cell death. Therefore, compounds with the ability to produce high levels of ROS and/or inhibit antioxidant processes are often used in anticancer therapies^35^. In the present study, the effect of **PCH-1** on ROS generation in A-549 cells was investigated by flow cytometry. As an indicator of ROS generation, the fluorescent probe 2ʹ,7ʹ-dichlorodihydrofluorescein diacetate (H2DCFDA) was used, which undergoes oxidation by ROS to form a highly fluorescent compound 2ʹ,7ʹ-dichlorofluorescein (DCF). As shown in Supplementary Fig. S6 in Supplementary Information, compound **PCH-1** induced a massive amount of ROS in A-549 cells in a time-dependent manner. During 3, 6, and 24 h was observed accordingly a significant 15% (****p < 0.00001), 56.9% (****p < 0.00001), and 68.1% (****p < 0.00001) increase of ROS towards vehicle control-treated cells. Moreover, the induction of ROS was equally high for **PCH-1** and the reference compound H_2_O_2_ in all the tested time points.

### Molecular docking studies

In addition to the abovementioned biological tests, a series of docking simulations were performed using the Autodock Vina program^36^. To estimate ligand–receptor affinity, an empirical scoring function inspired by the X-score function was used in Vina^37^. Docking was performed on 3 ligands, namely **PCH-1**, vinblastine (VLB), and vincristine (VNC), to investigate their binding mode in the vinblastine site of tubulin. The latter two ligands are already known for their effectiveness toward the tubulin target and served here as a reference^31^. Currently, there are a number of tubulin structures in the PDB database (such as 7DAD, 7DAE, 6KNZ, 6N47, and 6K9V). However, we decided that the most appropriate one for our study would be the 5JT2 structure from 2016 because it is a complex of protein with one of the ligands. Thus, we used the bound docking approach, for example, the structure of the target was used from the ligand–target complex. Both the receptor and the VLB ligand used in the docking simulation originated from the abovementioned 5J2T PDB file^38^. The VNC ligand was derived from PDB 7A69^39^. **PCH-1** is a new molecule; therefore, its structure was built and optimized using the Biovia software^40^. The maximum energy difference between the worst and the best docking modes was set to 5 kcal/mol. The grid center of Vina docking was selected as the center of mass of the ligand VLB from 5JT2, which was obtained using the experimental pose. The grid size in docking was set to 20 Å × 20 Å × 20 Å, which was adequate to cover the entire vinblastine site. Additionally, the rigid receptor and flexible ligands were parametrized by AutodockTools 1.5.6^41^. The parameterized systems were recorded in the PDBQT file. In particular, both receptor and ligand were presented using a united atom model, which involves the nonpolar hydrogen atoms. Atomic charges were estimated through the Gasteiger–Marsili method^42^.

The performed simulations were narrowed down to 10 best results based on the score function; after careful visual inspection, the best ones were selected based on their spatial arrangement and docking score (Table 4). The docking results indicate that **PCH-1** had scores similar to those of VLB, where the scores of VNC were lower by almost 2 kcal/mol, even with different spatial configurations. This finding agrees with our biological test results that **PCH-1** is biologically slightly more active than VLB or VNC.

**Table 4:** Ligand–protein energies calculated with the Autodock Vina program.

Ligands	ΔG (kcal/mol)
PCH-1	− 7.5
VLB	− 7.2
VNC	− 5.8

**Figure 8:**
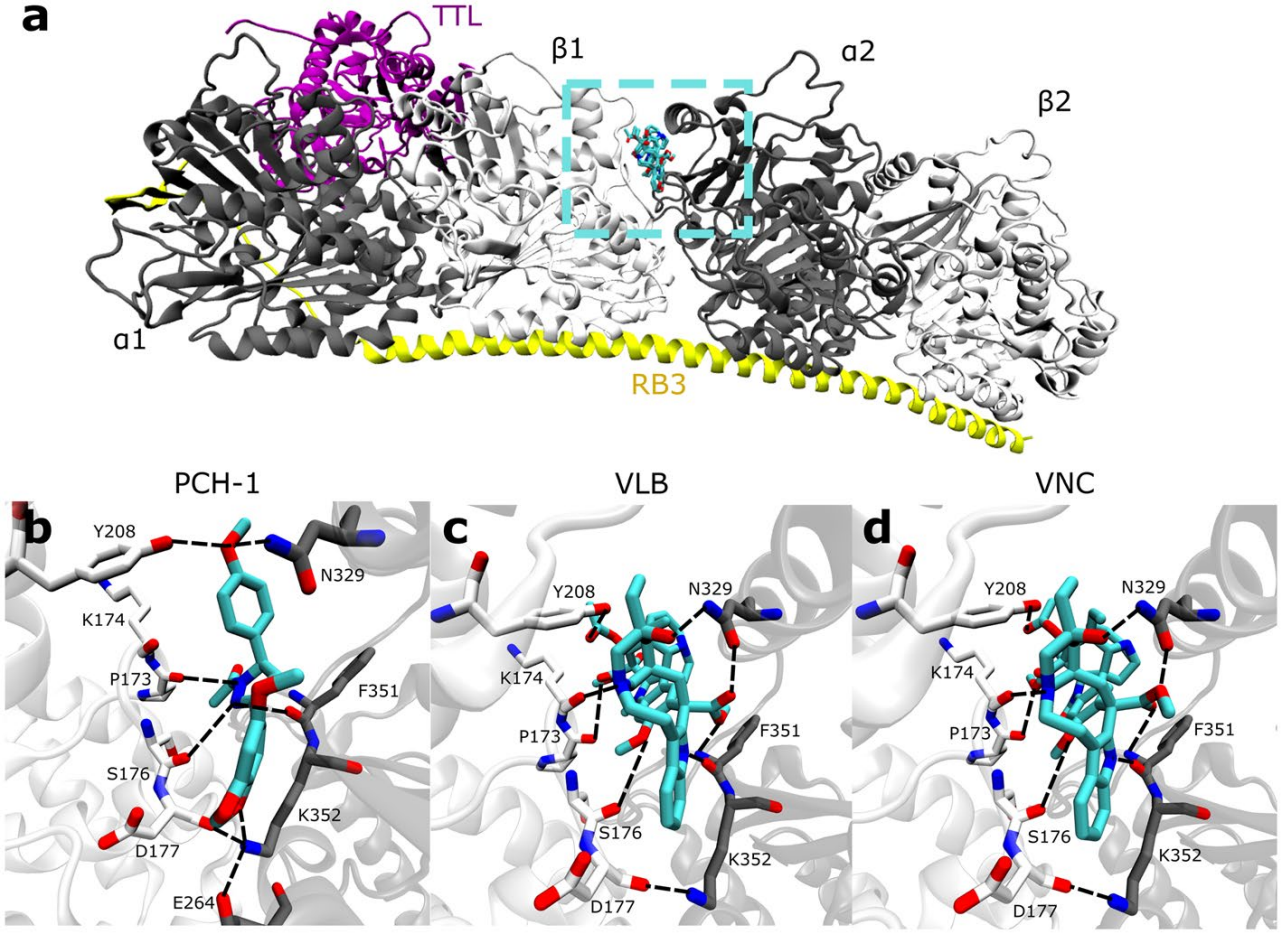
(**a**) Visualization of the complete tubulin protein in complex with TTL (purple) and RB3 (yellow); α tubulin (dark gray) and β tubulin (light gray) subunits were taken from PDB 5JT2. Additionally, PCH-1, VLB, and VNC (cyan) ligands docked at the vinblastine site of tubulin are marked with dashed lines; (**b–d**) Visualization of ligands at the docking site of PCH-1/VLB/VNC ligands, respectively, shown as licorice, where the carbon atoms are stained with cyan, oxygen with red, and nitrogen with blue. Tubulin β1 and α2 are displayed as light gray and dark gray bands, respectively. Our proposed receptor amino acids that may interact with the ligand are in the form of a stick and are labeled. The hydrogen bonds are underlined with black dashed lines.

These results were also visually checked by the VMD program^43^. Figure 8 was drawn using this program. Figure 8 a shows the complete structure of tubulin in a complex with TTL together with the α1, α2, β1, and β2 subunits. The additional box marked with dashed lines indicates the ligand docking site at the vinblastine site. Figure 8b–d are the magnifications of the mentioned place for each ligand. Each of them also shows the hydrogen bonds between the receptor amino acids and ligands; these bonds can hold the ligand in a permanent place and thus cause tubulin to malfunction. There was an apparent trend showing that the indole part attached to the heptylamine rings had a better score if it was located deeper at the vinblastine binding site of tubulin. This structure certainly influences the affinity of the ligand for the vinblastine site. It should be noted, however, that on the basis of molecular docking alone, it is difficult to conclude whether the proposed amino acids actually participate actively in the molecular mechanism of action. The main aim of performing molecular docking was to support biological data by checking whether the vinblastine binding site can be occupied by our ligands. More advanced molecular modeling methodology (thermodynamics study) could be used in future to further support our hypothesis about the binding site.

## Discussion

According to the World Health Organization (WHO), cancer is the leading cause of death worldwide and caused 10 million deaths in 2020, and lung cancer had the highest mortality rate and accounted for 1.8 million deaths^44^. The incidence of lung cancer is increasing dramatically each year, and this increase is associated with aging of the human population which has been exposed to several factors that promote mutations leading to cancer, such as smoking tobacco, unhealthy diet, or air pollution^60^. Treatment approaches for lung cancer mainly depend on tumor staging and type of cancer. Patients with small cell lung cancer show a good response to chemotherapy, while the progress in radiation therapy ensures their long-term survival^45^. Current pharmacological treatments for non-small cell lung cancer are not effective, but surgical resection offers substantial cure rates in early-stage cases. Patients with advanced, metastatic cancers are receiving combined chemotherapy and radiation^46^.

Many studies have proved that nitrogen-containing heterocyclic compounds such as pyrazole possess significant biological potency for cancer treatment^47,48^. In our present study, a series of pyrazoles derived from chalcones were reported based on the consideration of commonly used ring substitution modifications. Initially, the compounds were evaluated for cytotoxicity in the selected cell line models. Almost all derivatives exhibited moderate activity against all the three non-small cell lung cancer cell lines, except for the dimethoxy analogue **PCH-1**, which displayed potent cytotoxic properties and thus was chosen to evaluate its molecular mechanism of action. The substantial objective of cancer therapy is to kill cancer cells, but without affecting healthy cells. The toxicity studies on normal human embryonic kidney cells revealed that **PCH-1** is less toxic than the reference compound Cisplatin, and therefore, it is suitable for further evaluation of anticancer properties.

The determination of the mechanism responsible for cancer cell death is extremely important for drug efficacy and development. The antiproliferative effects of anticancer drugs are often linked with the suppression of the cell cycle. Studies on cell cycle distribution showed that **PCH-1** induces G_2_/M cell cycle arrest after 24 h of incubation in all the tested cancer cell lines.

Antimitotic properties of chalcones are known from 1990 and are associated with the inhibition of microtubule assembly^49^. Many existing microtubule inhibitors are registered as drugs, but their clinical use is often limited due to the chemoresistance mechanisms^50^. **PCH-1** inhibited tubulin polymerization in the cell-free tubulin assembly assay, but the effect was less pronounced similar to that observed for the reference compounds. In cell-based assays, we observed similar depolymerization of the tubulin network and impaired spindle assembly in the test compound and Vincristine groups. These results were supported by molecular docking.

Current cancer chemotherapies mostly exert an antitumor effect by triggering apoptosis in cells^51^. Apoptosis, also known as programmed cell death, is a natural mechanism for the regulation of physiological growth and homeostasis. This process is characterized by several morphological and biochemical hallmarks, including chromatin condensation, cell shrinkage, and phosphatidylserine externalization. There are also many ways in which cells may undergo apoptosis. The two main pathways linked to the activation of apoptosis signal transduction are intrinsic and extrinsic pathways^52^. Microscopic observations and flow cytometry analysis revealed that **PCH-1** induces apoptosis in all the tested cancer cell lines in a time-dependent manner. Therefore, one of the assumptions of our studies was to obtain information about the exact mechanism by which **PCH-1** induces apoptosis. For this purpose, the A-549 cell line was selected as the most sensitive cell line to the test compound. Western blot analyses revealed full activation of the initiation caspase-9 and execution caspase-3, with a significant increase in their cleaved forms in a time-dependent manner. Caspase-9 is activated by translocation from the cytosol into the intermembrane space and partially by recruitment into the Apaf-1 apoptosome complex following the efflux of cytochrome *c* from the mitochondria^53^. Once activated, caspase-9 cleaves and activates caspase-3 and caspase-7 to induce apoptosis^54^. Next, the activated caspases cleave cellular proteins such as nuclear enzyme PARP to dismantle the apoptotic cells^55^. Cleavage and inactivation of PARP by **PCH-1** lead to loss of genomic integrity, thereby depleting the cell of NAD and ATP and thus contributing to cell death. Moreover, the activation of caspase-8 was also observed in **PCH-1-**treated cells, which is closely related to apoptosis signaling by the extrinsic pathway^56^. Caspase-8 mediates the mitochondrial pathways through the cleavage of the proapoptotic Bcl-2 family members such as Bid to tBid. Subsequently, tBid switches the conformation of Bak, resulting in its oligomerization and allosteric activation and releasing cytochrome *c*^57^. **PCH-1** treatment leads to both cleavage of Bid and increased expression of Bax, thus suggesting the activation of cell surface death receptors and therefore the extrinsic apoptosis pathway.

Metastasis involves the spread of cancer cells from the primary tumor site to other tissues or organs of the body and is the major cause of cancer-related deaths^58^. One of the earliest events in cancer metastasis is EMT, a process that converts epithelial cells to mesenchymal cells that undergo cytoskeletal rearrangements to promote cancer motility and invasiveness^59^. Intermediate filaments (IFs) play an important role in EMT progression by maintaining cellular polarity and stiffness^60^. The most abundant member of the IF family is Vimentin (a type III protein) that is responsible for forming associated parallel arrays with microtubules and modulating prosurvival functions^61^. The disassembling of the microtubule network leads to a collapse of Vimentin IFs to the perinuclear region^62^. Recent studies have shown the significance of Vimentin in lung cancer, wherein a high expression of Vimentin was found to be related to a high frequency of metastases and therefore a poor prognosis in patients with resected non-small cell lung cancer^63^. On the basis of these findings, we decided to test the effect of **PCH-1** on the suppression of the EMT process. **PCH-1** effectively downregulated Vimentin and significantly promoted the suppression of another mesenchymal marker, *N*-cadherin, in A-549 cells. In order to show retrieving the epithelial phenotypes capacity by tested compound, A-549 cells were stimulated with well-known EMT inducer TGF-β1, and then exposed to **PCH-1**^64^. Treatment with TGF-β1 alone activated *N*-cadherin, and β-catenin, whereas co-treatment with **PCH-1** abrogated their expression. This suggests that **PCH-1** is a therapeutic agent for cancer treatment controlling TGF-β1-mediated EMT. Moreover, we studied the expression of telomerase reverse transcriptase, which plays a key role in ensuring chromosomal stability by maintaining telomere length, and therefore assert the uncontrolled proliferation of the cells^65^. Besides, telomerase exhibit many multiple non-canonical biological functions, like protecting mitochondrial DNA, regulating the expression of Wnt target genes (serving a critical role in tumorigenesis), and promoting EMT through the NF-kB signaling pathway^66–68^. Additionally, Liu et al. shows that depletion of hTERT leads to abolished TGF-β1 and β-catenin-mediated EMT. β-catenin in cooperation with hTERT regulates transcription of Vimentin, so deregulation of all those proteins leads to preventing cancer progression^69^. We demonstrated that **PCH-1** significantly decreased the level of hTERT, which suggests that it not only suppresses EMT but also inhibits tumor proliferation and induces cell mortality.

Microtubule dynamics are essential in the cell migration by providing protrusive and contractile forces, a front-rear polarity allowing movement of organelle and protein through the cells, and controlling signaling during motility^70,71^. Many microtubule-targeting agents inhibit cell migration, by preventing activation of Rac1/Cdc42^72,73^. During microtubule polymerization, Rac1 is activated in lamellipodia and stimulates actin stress fiber formation. Whereas, Cdc42 regulates polarization of cells, and proper orientation of the centrosome. Consequently, interference with microtubule dynamics inhibit Rac1 and Cdc42, and therefore decrease cell motility. **PCH-1** efficiently decreased cell migration in concentration-dependent manner in TGF-β1-stimulated, and unstimulated cells, which was observed under time-lapse microscopy by using a wound healing assay. However, the exact mechanism of inhibition cell migration by **PCH-1** should be further explored, such as confirming the involvement of Rac1 and Cdc42 in this process.

Several studies have shown that many compounds derived from chalcone possess oxidative properties^74,75^. Similar to its analogs, **PCH-1** robustly induces the generation of ROS in A-549 cells. This accumulation can activate both the intrinsic and extrinsic apoptotic cell death, by activating the downstream caspase cascades^76^.

**Figure 9:**
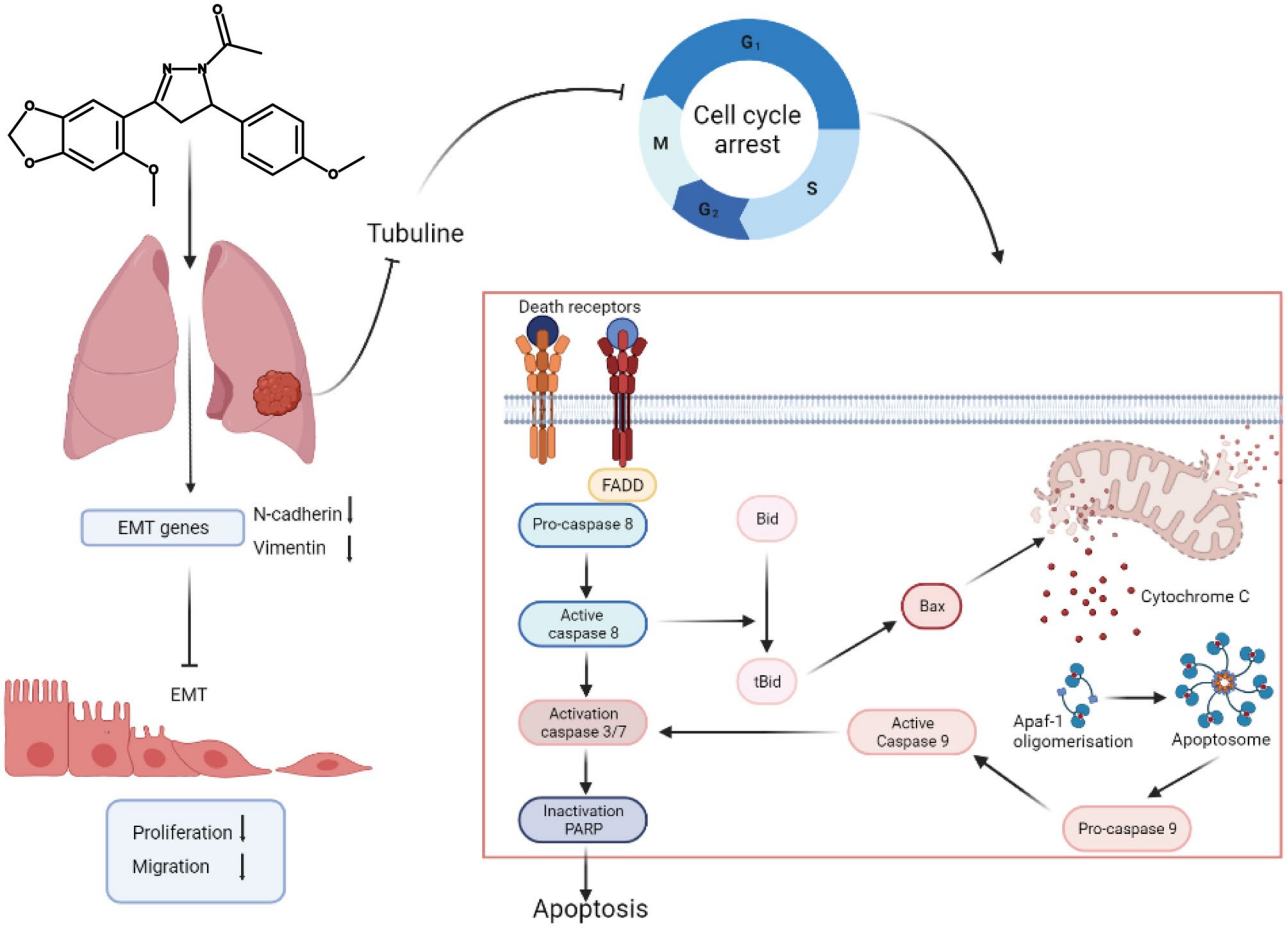
Molecular mechanism of action **PCH-1** compound against lung cancer cells.

In conclusion, this study provides evidence that **PCH-1** reduces cell viability and colony formation of non-small lung cancer cells in vitro. Our study demonstrated that **PCH-1** induces G_2_/M phase arrest by stabilizing tubulin polymers and cellular apoptosis through extrinsic and intrinsic pathways. In addition to its proapoptotic properties, **PCH-1** shows potent antimetastatic and prooxidant potential in A-549 cells. The molecular mechanism of action of this compound is shown in Fig. 9. On the basis of the presented findings, **PCH-1** is a promising antitumor compound that warrants further preclinical development.

## Methods

### Synthesis of the designed compounds

Liquid chromatography-mass spectrometry (LCMS) spectra were registered on an LCMS IT-TOF (Shimadzu, Japan) with an electrospray ionization (ESI) source in the positive ion mode. ^1^H-NMR spectra were acquired on a Bruker Avance III spectrometer at 500 MHz. The NMR data were processed and analyzed by TopSpin (Bruker, USA). Chemical shifts were expressed at δ value to the internal standard (TMS). IR spectra were measured on an IF S66 spectrometer (Bruker, USA) in KBr pellets at an absorption range of 400–4000 cm^−1^. Thin layer chromatography (TLC) was performed on Merck Kieselgel 60 F254 aluminum plates and visualized with UV and iodine.

Each compound was synthesized in two stages: condensation of the appropriately substituted benzaldehydes and acetophenones, and reaction with hydrazine hydrate to yield chalcone-derived pyrazoles.

### General procedure for the synthesis of chalcones

First, 10 mmol of the appropriately substituted benzaldehyde and acetophenone were dissolved in 20 ml of ethanol. The mixture was then stirred at 5 °C for few minutes. Next, 10 ml of 40% aqueous KOH was added, and the solution was stirred at room temperature (RT) for approximately 4 h. The formed precipitate was collected by filtration, characterized by HPLC and LCMS, and subjected to further reaction.

### General procedure for the synthesis of chalcone-derived pyrazoles

Hydrazine hydrate (20 mmol) was added to a solution of chalcone (5 mmol) in acetic acid (10 ml). The reaction mixture was refluxed for approximately 2 h. The progress of the reaction was monitored by TLC. After the completion of the reaction, the mixture was cooled, adjusted to pH 7 with 10% Na_2_CO_3_, poured into crushed ice, and allowed to stand overnight at RT. The product was collected by filtration and purified by flash chromatography or reversed-phase high-performance liquid chromatography (RP-HPLC). The product was then characterized by LCMS, ^1^H-NMR, and IR.

#### 1-[3-(6-methoxy-2*H*-1,3-benzodioxol-5-yl)-5-(4-methoxyphenyl)-4,5-dihydro-1*H*-pyrazol-1-yl]ethan-1-one (PCH-1)

ESI–MS: (*m/z*): 369.139 [M + H]^+^; ^1^H-NMR (CDCl_3_, 500 MHz), δ (ppm): 2.39 (s, 3H), 3.30 (dd, *J*_*1*_ = 3.77 Hz, *J*_*2*_ = 3.77 Hz, 2H), 3.78 (s, 3H), 3.79 (s, 3H), 5.49 (dd, *J*_*1*_ = 3.23 Hz, *J*_*2*_ = 3.41 Hz, 1H), 6.00 (s, 2H), 6.54 (s, 1H), 6.85 (d, *J* = 8.26 Hz, 2H), 7.19 (d, *J* = 7.89 Hz, 2H), 7.48 (s, 1H); IR (KBr), ν (cm^−1^): 1650.92, 1468.94.

#### 1-[5-(2*H*-1,3-benzodioxol-5-yl)-3-(3,4,5-trimethoxyphenyl)-4,5-dihydro-1*H*-pyrazol-1-yl]ethan-1-one (PCH-2)

ESI–MS: (*m/z*): 399.125 [M + H]^+^; ^1^H-NMR (CDCl_3_, 500 MHz), δ (ppm): 2.45 (s, 3H), 3.14 (dd, *J*_*1*_ = 4.31 Hz, *J*_*2*_ = 4.30 Hz, 1H), 3.73 (dd, *J*_*1*_ = 11.84 Hz, *J*_*2*_ = 11.85 Hz, 1H), 3.91 (s, 3H), 3.93 (s, 6H), 5.54 (dd, *J*_*1*_ = 4.31 Hz, *J*_*2*_ = 4.66 Hz, 1H), 5.94 (s, 2H), 6.73 (m, 3H), 6.97 (s, 2H); IR (KBr), ν (cm^−1^): 1665.00, 1417.26.

#### 1-[5-(2*H*-1,3-benzodioxol-5-yl)-3-(4-methylphenyl)-4,5-dihydro-1*H*-pyrazol-1-yl]ethan-1-one (PCH-3)

ESI–MS: (*m/z*): 323.098 [M + H]^+^; ^1^H-NMR (CDCl_3_, 500 MHz), δ (ppm): 2.42 (s, 3H), 2.44 (s, 3H), 3.15 (d, *J* = 16.59 Hz, 1H), 3.73 (dd, *J*_*1*_ = 10.78 Hz, *J*_*2*_ = 11.19 Hz, 1H), 5.52 (d, *J* = 7.05 Hz, 1H), 5.94 (s, 2H), 6.70 (s, 1H), 6.75 (s, 2H), 7.25 (d, *J* = 7.46 Hz, 2H), 7.65 (d, *J* = 7.88 Hz, 2H); IR (KBr), ν (cm^−1^): 1649.10, 1443.97.

#### 1-[5-(2*H*-1,3-benzodioxol-5-yl)-3-(2-methoxyphenyl)-4,5-dihydro-1*H*-pyrazol-1-yl]ethan-1-one (PCH-4)

ESI–MS: (*m/z*): 339.101 [M + H]^+^; ^1^H-NMR (CDCl_3_, 500 MHz), δ (ppm): 2.42 (s, 3H), 3.31 (dd, *J*_*1*_ = 4.27 Hz, *J*_*2*_ = 4.60 Hz, 1H), 3.85 (s, 3H), 3.86 (dd, *J*_*1*_ = 11.50 Hz, *J*_*2*_ = 11.83 Hz, 1H), 5.47 (dd, *J*_*1*_ = 4.60 Hz, *J*_*2*_ = 4.60 Hz, 1H), 5.93 (s, 2H), 6.73 (s, 1H), 6.76 (s, 2H), 6.96 (d, *J* = 8.21 Hz, 1H), 7.04 (t, *J*_*1*_ = 7.56 Hz, *J*_*2*_ = 7.32 Hz, 1H), 7.41 (m, 1H), 7.94 (dd, *J*_*1*_ = 1.64 Hz, *J*_*2*_ = 1.81 Hz, 1H); IR (KBr), ν (cm^−1^): 1659.49, 1419.84.

#### 1-[3,5-bis(2*H*-1,3-benzodioxol-5-yl)-4,5-dihydro-1*H*-pyrazol-1-yl]ethan-1-one (PCH-5)

ESI–MS: (*m/z*): 353.068 [M + H]^+^; ^1^H-NMR (CDCl_3_, 500 MHz), δ (ppm): 2.42 (s, 3H), 3.09 (dd, *J*_*1*_ = 4.61 Hz, *J*_*2*_ = 4.23 Hz, 1H), 3.68 (dd, *J*_*1*_ = 11.53 Hz, *J*_*2*_ = 11.53 Hz, 1H), 5.51 (dd, *J*_*1*_ = 4.62 Hz, *J*_*2*_ = 4.23 Hz, 1H), 5.94 (s, 2H), 6.05 (s, 2H), 6.69 (s, 1H), 6.76 (m, 2H), 6.85 (d, *J* = 8.57 Hz, 1H), 7.11 (dd, *J*_*1*_ = 1.54 Hz, *J*_*2*_ = 1.54 Hz, 1H), 7.39 (d, *J* = 1.53 Hz, 1H); IR (KBr), ν (cm^−1^): 1653.92, 1458.82.

#### 1-{5-(2*H*-1,3-benzodioxol-5-yl)-3-[4-(trifluoromethoxy)phenyl]-4,5-dihydro-1*H*-pyrazol-1-yl}ethan-1-one (PCH-6)

ESI–MS: (*m/z*): 393.056 [M + H]^+^; ^1^H-NMR (CDCl_3_, 500 MHz), δ (ppm): 2.44 (s, 3H), 3.14 (dd, *J*_*1*_ = 4.40 Hz, *J*_*2*_ = 4.83 Hz, 1H), 3.73 (dd, *J*_*1*_ = 11.86 Hz, *J*_*2*_ = 11.86 Hz, 1H), 5.55 (dd, *J*_*1*_ = 4.40 Hz, *J*_*2*_ = 4.39 Hz, 1H), 5.94 (s, 2H), 6.73 (m, 3H), 7.29 (d, *J* = 8.95 Hz, 2H), 7.79 (d, *J* = 8.63 Hz, 2H); IR (KBr), ν (cm^−1^): 1652.13, 1447.62.

#### 1-[5-(2,3-dihydro-1-benzofuran-5-yl)-3-(4-methoxyphenyl)-4,5-dihydro-1*H*-pyrazol-1-yl]ethan-1-one (PCH-7)

ESI–MS: (*m/z*): 337.108 [M + H]^+^; ^1^H-NMR (CDCl_3_, 500 MHz), δ (ppm): 2.43 (s, 3H), 3.16 (m, 3H), 3.71 (dd, *J*_*1*_ = 11.79 Hz, *J*_*2*_ = 11.79 Hz, 1H), 3.88 (s, 3H), 4.55 (t, *J*_*1*_ = 8.84 Hz, *J*_*2*_ = 8.42 Hz, 2H), 5.53 (dd, *J*_*1*_ = 4.42 Hz, *J*_*2*_ = 4.21 Hz, 1H), 6.73 (d, *J* = 8.00 Hz, 1H), 6.96 (d, *J* = 8.85 Hz, 2H), 7.01 (dd, *J*_*1*_ = 1.47 Hz, *J*_*2*_ = 1.48 Hz, 1H), 7.07 (s, 3H), 7.72 (d, *J* = 8.85 Hz, 2H); IR (KBr), ν (cm^−1^): 1661.06, 1493.03.

#### 1-[5-(6-methoxy-2*H*-1,3-benzodioxol-5-yl)-3-(4-methoxyphenyl)-4,5-dihydro-1*H*-pyrazol-1-yl]ethan-1-one (PCH-8)

ESI–MS: (*m/z*): 369.092 [M + H]^+^; ^1^H-NMR (CDCl_3_, 500 MHz), δ (ppm): 2.46 (s, 3H), 2.98 (dd, *J*_*1*_ = 4.40 Hz, *J*_*2*_ = 4.41 Hz, 1H), 3.67 (dd, *J*_*1*_ = 11.64 Hz, *J*_*2*_ = 11.63 Hz, 1H), 3.82 (s, 3H), 3.86 (s, 3H), 5.77 (dd, *J*_*1*_ = 4.40 Hz, *J*_*2*_ = 4.41 Hz, 1H), 5.88 (s, 2H), 6.54 (d, *J* = 5.66 Hz, 2H), 6.93 (d, *J* = 8.80 Hz, 2H), 7.68 (d, *J* = 8.80 Hz, 2H); IR (KBr), ν (cm^−1^): 1661.13, 1483.86.

#### 1-[5-(4-hydroxyphenyl)-3-(6-methoxy-2*H*-1,3-benzoxathiol-5-yl)-4,5-dihydro-1*H*-pyrazol-1-yl]ethan-1-one (PCH-9)

ESI–MS: (*m/z*): 371.045 [M + H]^+^; ^1^H-NMR (CDCl_3_, 500 MHz), δ (ppm): 2.32 (s, 3H), 3.18 (dd, *J*_*1*_ = 4.33 Hz, *J*_*2*_ = 4.33 Hz, 1H), 3.69 (s, 3H), 3.73 (dd, *J*_*1*_ = 7.11 Hz, *J*_*2*_ = 8.39 Hz, 1H), 5.37 (dd, *J*_*1*_ = 4.33 Hz, *J*_*2*_ = 4.33 Hz, 1H), 5.67 (s, 2H), 6.42 (s, 1H), 6.55 (d, *J* = 8.66 Hz, 2H), 6.94 (d, *J* = 8.66 Hz, 2H), 7.69 (s, 1H); IR (KBr), ν (cm^−1^): 1638.59, 1468.15.

### Biological evaluation

#### Cell culture

HEK293, A-549, H226, and H460 cells were obtained from the American Type Culture Collection (ATCC, USA) and cultured in DMEM (HEK293) or RPMI medium (Corning) supplemented with 10% fetal bovine serum (Corning), 2 mM l-glutamine (Sigma-Aldrich), and antibiotics (penicillin 62.6 µg/ml and streptomycin 40 µg/ml, Sigma-Aldrich). The cells were incubated in a humidified 5% CO_2_ atmosphere at 37 °C and routinely screened for *Mycoplasma* contamination.

#### Cell viability assay

The cell viability assay was performed for A-549, H226, H460, and HEK293 cells after treatment with the test compounds by using the 3-(4,5-dimethylthiazol-2-yl)-2,5-diphenyltetrazolium bromide (MTT, Sigma-Aldrich) assay. Briefly, cells were seeded onto 96-well culture plates for attachment. After 24 h, triplicate wells were treated with DMSO (1%) and the agents. After 72 h of incubation at 37 °C in 5% CO_2_, the MTT solution (0.4 mg/ml) was added to each well. After 2–4 h of incubation, the medium was removed, and the formazan crystals were dissolved in 100 μl of DMSO. The absorption was measured at 450 nm with an ASYS UVM340 microplate reader (Biochrom Ltd.). The 50% inhibitory concentration (IC_50_) was defined as the concentration that reduced the absorbance of the DMSO-treated wells by 50% of the vehicle. The IC_50_ values were generated from GraphPad Prism 9 software by plotting the survival curve as a function of dose from 3 independent experiments***.***

#### Colony formation assay

Cells were seeded onto 6-well plates (500 cells/well) and allowed to attach for 24 h in a fresh RPMI medium. Next, the cells were pretreated with DMSO (1%) solution of the examined compound (at the concentrations of 1, 5, and 10 µM) for 24 h. The medium containing compound was then changed, and the cells were cultured for additional 9 days. After incubation, the cells were washed twice with phosphate-buffered saline (PBS), fixed with 100% methanol for 30 min, and stained with 0.5% crystal violet for 15 min. The colonies were photographed and counted by ImageJ software.

#### Morphology assessment

Cells were seeded onto tissue culture plates with a glass slide and allowed to attach for 24 h. Next, the cells were treated for the indicated time with **PCH-1** or DMSO. After incubation, the cells were rinsed with PBS, fixed in 3.7% paraformaldehyde (Sigma-Aldrich) for 10 min at RT and stained with 4′,6-diamidino-2-phenylindole (DAPI, Sigma-Aldrich). The stained nuclei were visualized using a fluorescence microscope (Olympus BX60) with an appropriate filter***.***

#### Analysis of cell cycle distribution

Cells were seeded onto tissue culture plates and allowed to attach for 24 h. Unsynchronized cells were treated with **PCH-1** or Etoposide at IC_90_ concentration for 24 and 48 h, while synchronized cells were treated with **PCH-1** or Etoposide at IC_90_ concentration for 2, 4, 6, 8, and 10 h. Synchronization of the cells was performed as described previously by Chen et al.^77^. Next, the cells were trypsinized, fixed in ice-cold 75% ethanol, and stored overnight at − 20 °C. After centrifugation, the cells were rinsed with PBS and stained with 20 μg/μl PI (Sigma-Aldrich) and 50 μg/μl RNaseA (Thermo Fisher) in PBS for 30 min at RT. The distribution of the cells in different phases of the cell cycle was determined by flow cytometry (Guava EasyCyte 8 cell sorter, Merck Millipore) and FlowJo software v10. Each experiment was repeated independently three times.

#### HTS-tubulin polymerization assay biochem kit

The assay was performed according to the manufacturer’s instructions. Briefly, compounds were diluted in DMSO to a concentration of 2 mM and then in GTB buffer to a concentration of 50 µM. In the wells of a 96-well plate (UV transparent, half surface wells) previously heated to 37 °C, 10 µl of dilutions of the test compounds were placed in duplicate. The plate was incubated for 2 min at 37 °C, and 100 µl of freshly prepared tubulin solution (4 mg/ml) in GTB-GTP buffer was then added. The plate was immediately placed in a Tecan SPARK 10 M plate reader, and the reaction kinetics were measured with the following settings: absorbance reading: 340 nm, 37 °C, interval: 45 s, number of cycles: 160.

#### Immunofluorescence

Cells were seeded onto tissue culture plates with a glass slide and allowed to attach for 24 h. After the incubation period, the cells were washed in PBS, fixed for 15 min at RT with 4% paraformaldehyde (Sigma-Aldrich) in PBS, and permeabilized for 15 min in 0.25% Triton-X100 (Sigma-Aldrich) in PBS. The cells were then washed twice with PBS, blocked with 3% bovine serum albumin (BSA, Sigma-Aldrich) in PBS for 1 h at RT, and incubated for 1 h at 37 °C in a humidified chamber with the following primary antibodies diluted in 3% BSA in PBS-T (PBS with 0.1% (v/v) Triton X-100): rabbit anti-Aurora B antibody (Abcam, ab2254) 1:500 and mouse anti-Tubulin 1:200 (Sigma-Aldrich, T8328). The slides were washed three times with PBS-T and incubated for 1 h at 37 °C in a humidified chamber with the following secondary antibodies diluted in 3% BSA in PBS-T: goat anti-mouse IgG antibody conjugated with Fluorochrome DyLight-488 (1:250, Thermo Fisher) and goat anti-rabbit IgG antibody conjugated to Alexa Fluor 594 (1:200, Jackson ImmunoResearch). The slides were then washed twice in PBS-T and stained with 0.25 µg/ml DAPI. Images were acquired with an LSM 800 inverted laser scanning confocal microscope (Carl Zeiss) with an Airyscan detector using a × 63 1.4 NA Plan Apochromat objective (Carl Zeiss).

#### Apoptosis and caspase 3/7 assay

Cells were seeded onto tissue culture plates and allowed to attach for 24 h. Next, the cells were treated with **PCH-1** or Etoposide at IC_90_ concentration for 6, 24, and 48 h. After incubation, the cells were harvested by trypsinization, rinsed twice with PBS, and stained with Annexin V Alexa Fluor™ 488 conjugate (Thermo Fisher, #A13201) for apoptosis assay and with CellEvent™ Caspase-3/7 Green Flow Cytometry Assay Kit (Thermo Fisher, #C10427) for caspase-3/7 activation according to the manufacturer’s protocols. The analysis was performed with Guava EasyCyte 8 cell sorter (Merck Millipore) and FlowJo software v10. Each experiment was repeated independently three times.

#### Western blot

Cells (1.5 × 10^6^) were cultured in tissue culture plates for overnight attachment. The cells were then incubated with IC_90_ of **PCH-1** or 1% DMSO for 6, 24, and 48 h, or were co-treatment with 2.5 ng/ml TGF-β1 (Sigma-Aldrich), and IC_90_ of PCH-1 or 1% DMSO for 24, and 48 h. Subsequently, the cells were collected and lysed in Laemmli buffer with a protease inhibitor cocktail (Roche) and phosphatase inhibitors [NaF (5 mM), β-glycerophosphate (5 mM), and Na_3_VO_4_ (1 mM)]. The cells were then sonicated in triplicate (10 s, 30% amplitude) and centrifuged at 16,000×*g* at 12 °C for 15 min. Protein concentrations were determined using the DC Protein Assay Kit (Bio-Rad). For determining total protein content, the cellular extract was separated by 10% or 12% sodium dodecyl sulfate–polyacrylamide gel electrophoresis (SDS-PAGE) and transferred onto a microporous polyvinylidene difluoride (PVDF) membrane (Bio-Rad). After blocking with 5% (w/v) BSA (Sigma-Aldrich) in 1 × TBST (pH 7.4 and 0.1% Tween-20) for 1 h, the membranes were incubated overnight with primary antibodies at 4 °C, followed by horseradish peroxidase-conjugated secondary antibodies for 1 h. All the used antibodies are listed in Table S1 in the supplementary information. The immunoreactive signals were detected using an enhanced chemiluminescence (ECL) detection reagent kit (Thermo Fisher) and a ChemiDoc XRS + Imaging System (Bio-Rad). The band intensity was measured using Image Lab 5.2 software (Bio-Rad).

#### Wound healing migration assay

To analyze cell motility, A-549 cells were seeded onto the Ibidi-silicone insert on a cover glass-bottom 24-well plate for live cell imaging and incubated for 24 h. Subsequently, the inserts were dislodged, the cellular debris was removed by washing with RPMI, and the cells were incubated with different concentrations of **PCH-1,** and/or 2.5 ng/ml TGF-β1 (Sigma-Aldrich) in an imaging chamber (cellVivo incubation system, Olympus) at 37 °C with 5% CO_2_. Images were captured every 10 min for 20 or 42 h under × 10 magnification using a fluorescence microscope (IX83 Inverted Microscope, Olympus) connected to an XC50 digital color camera (Olympus). The percentage of wound closure was quantified with ImageJ software.

#### Intracellular ROS measurement

For determination the ROS generation, cells were treated with IC90 of **PCH-1,** H_2_O_2,_ or 1% DMSO for 3, 6, and 24 h. Next, a 1 µM CM-H_2_DCFDA (#C6827; Thermo-Fisher) probe was added to the cells in each plate 30 min before the end of the drug treatment. After incubation cells were collected by trypsinization, stained with 7-Aminoactinomycin D (7-AAD, Sigma-Aldrich), and measured. The analysis was performed with Guava EasyCyte 8 cell sorter (Merck Millipore) and FlowJo software v10. Each experiment was repeated independently three times.

## Supplementary Information

Supplementary Information.
