# Supplementary material for: Novel chalcone-derived pyrazoles as potential therapeutic agents for the treatment of non-small cell lung cancer
Source: Sci Rep. 2022 Mar 8;12:3703. doi: 10.1038/s41598-022-07691-6 (PMC8904451; doi:10.1038/s41598-022-07691-6)
Supplement: Supplementary file 1 — Supplementary Information. [file 41598_2022_7691_MOESM1_ESM.docx]

***Supplementary Information***

***Novel Chalcone-derived Pyrazoles as Potential Therapeutic Agents for the Treatment of Non-small Cell Lung Cancer***

Natalia Maciejewska^1*^, Mateusz Olszewski^1^, Jakub Jurasz^1^, Marcin Serocki^3^, Maria Dzierzynska^2^, Katarzyna Cekala^2^, Ewa Wieczerzak^2^, Maciej Baginski^1^

*^1^ Faculty of Chemistry, Gdansk University of Technology, Gabriela Narutowicza 11/12, 80-233 Gdansk, Poland*

*^2^ Faculty of Chemistry, University of Gdansk, Wita Stwosza 63, 80-308 Gdansk, Poland*

*^3^Ryvu Therapeutics, Leona Henryka Sternbacha 2, 30-394 Krakow, Poland*

*Corresponding authors: natalia.maciejewska@pg.edu.pl

**Biological evaluation**


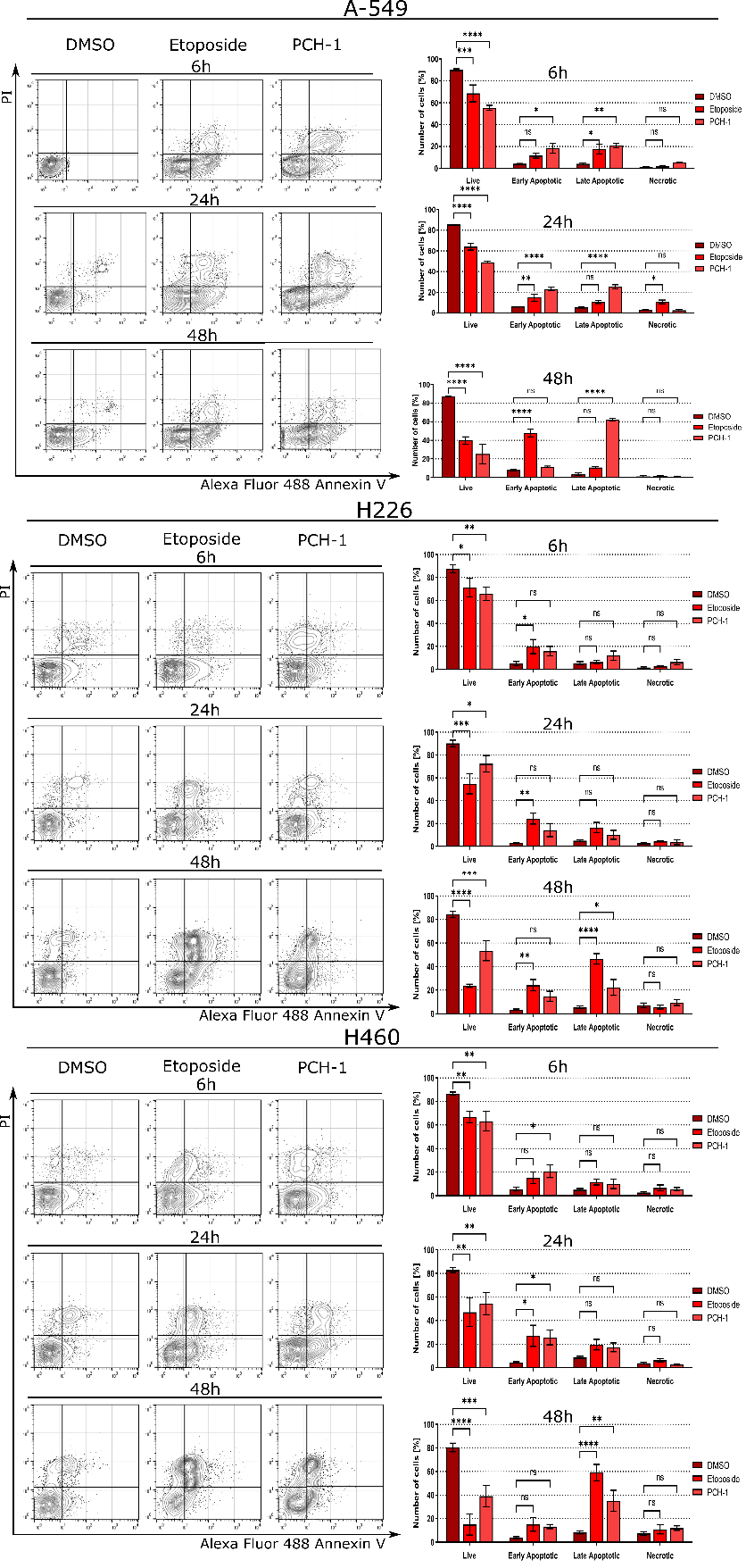


**Figure S1** Flow cytometric analyses of A-549, H226, and H460 cells after Alexa Fluor 488 Annexin V /PI staining. Representative dot-plots at 6, 24, and 48 h of treatment with **PCH-1**, Etoposide, or DMSO are presented on the left side. The quantitation of analysis is presented on a bar graph, on the right side. Error bars represent the SEM of data obtained in n=3 independent experiments. Statistical differences were analyzed with one-way ANOVA and post hoc Dunnet’s test. ^ns^ p>0.05, * p<0.01, ** p<0.001, *** p<0.0001, **** p<00001 vs. vehicle.


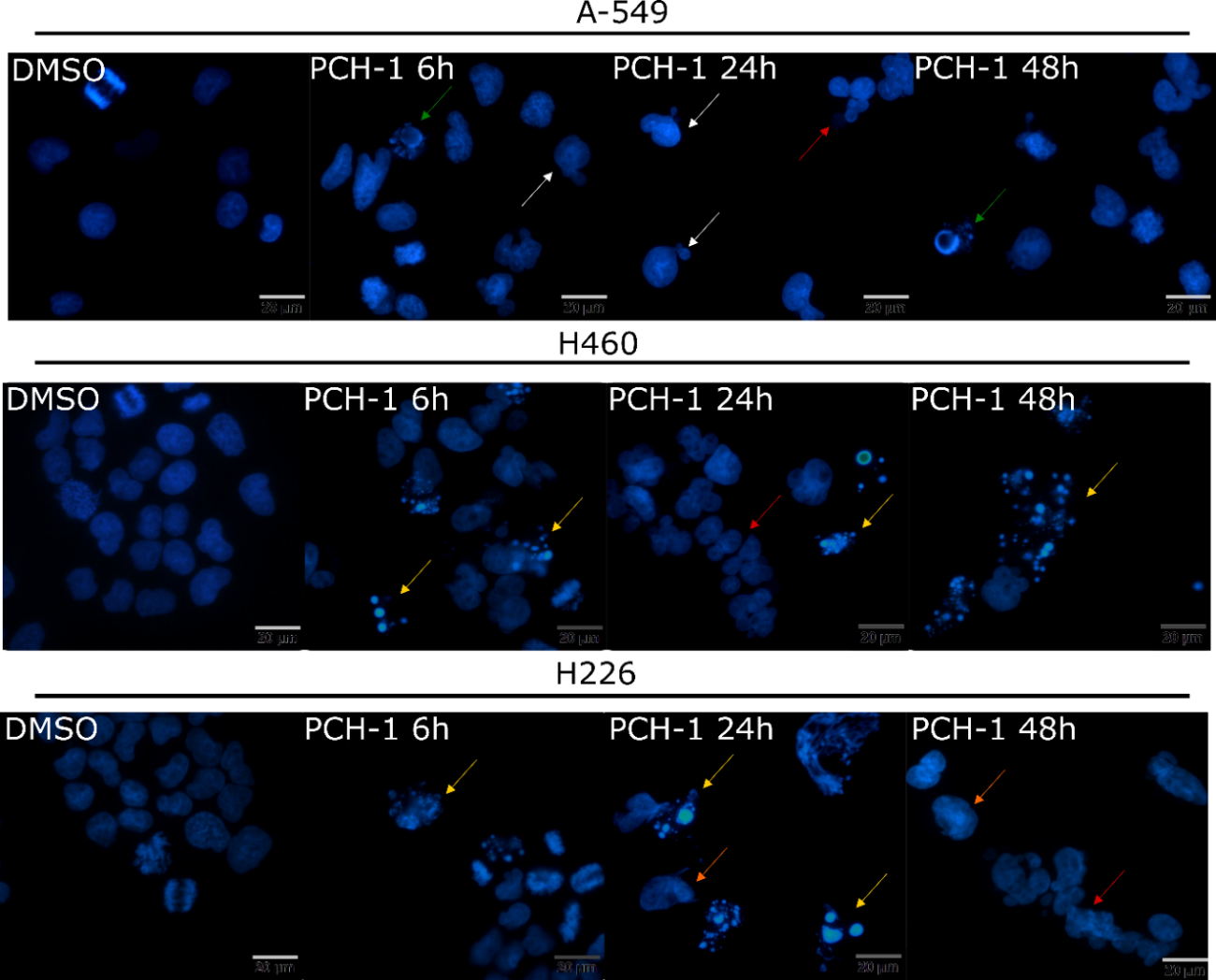


**Figure S2** Fluorescence images of A-549, H226, and H460 cells at 6, 24, and 48 h of treatment with **PCH-1** or DMSO. The cells were stained with DAPI and visualized by fluorescence microscopy, scale bar = 20 µM. The arrows indicate cell blebbing (white), cell shrinkage (green), polyploid cells (red), apoptotic bodies (yellow), and chromatin condensation (orange).

To confirm proapoptotic properties of investigated compound, A-549, H226, and H460 cells were treated with the **PCH-1** for 6, 24, and 48 h, and their nuclei were stained with DAPI and visualized by fluorescent microscopy**.** As depicted in **Figure S2**, **PCH-1** treatment exhibited typical phenotypic features of cells undergoing apoptosis, as evidenced by chromatin condensation, cell shrinkage, and membrane blebbing. Increasing the time of exposure to **PCH-1** triggered the formation of multinucleated polyploid cells, which is likely due to abnormalities of the spindle.


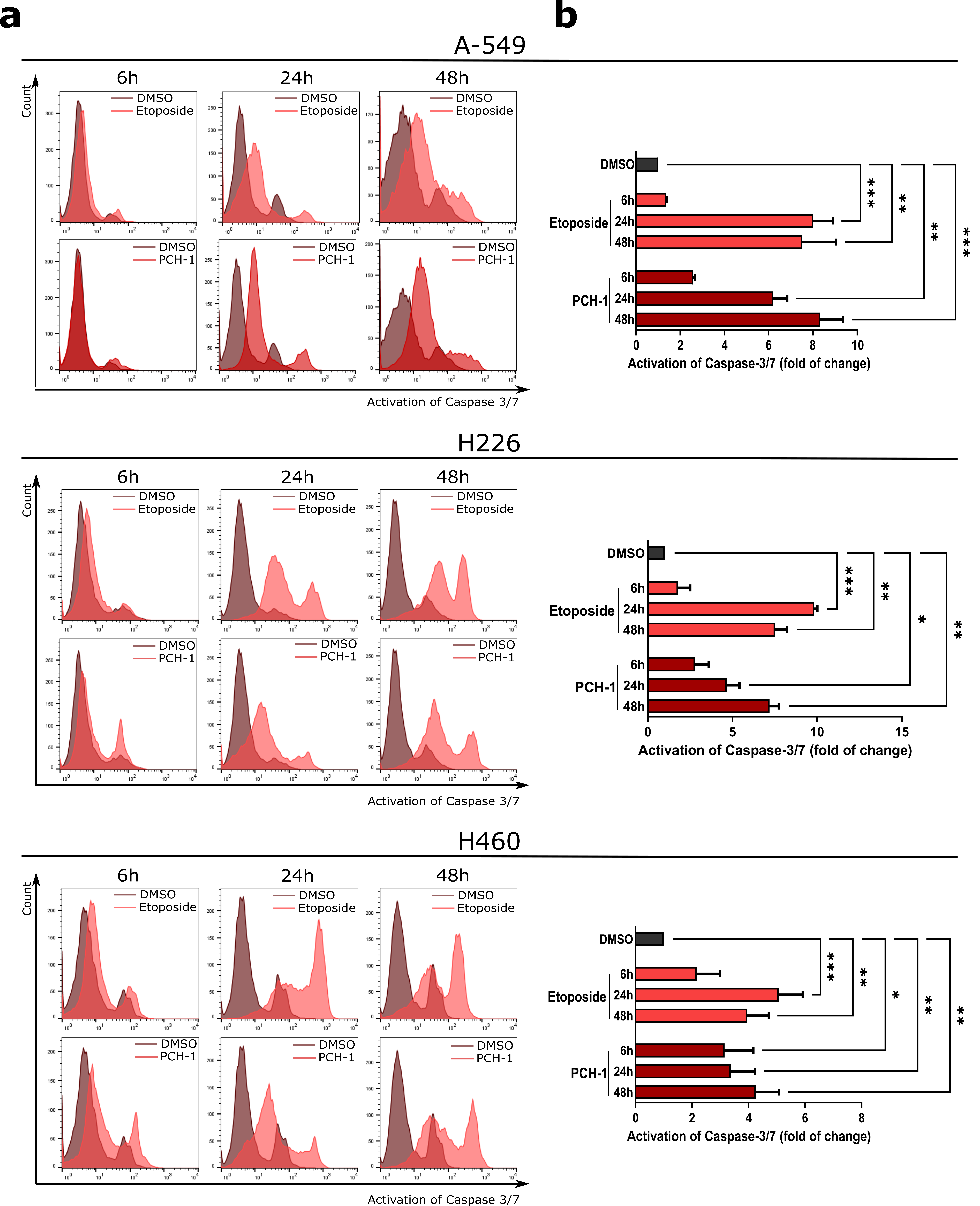


**Figure S3** Flow cytometric analyses of caspase-3/7 activation in A-549, H226, and H460 cell lines at 6, 24, and 48 h of treatment. **a.** Representative histograms; **b.** The quantitation of analysis is presented on a bar graph. Data represent mean ± SEM of three independent experiments. Statistical differences were analyzed by one-way ANOVA and post hoc Dunnett`s test. * p<0.01, ** p<0.001, *** p<0.0001.


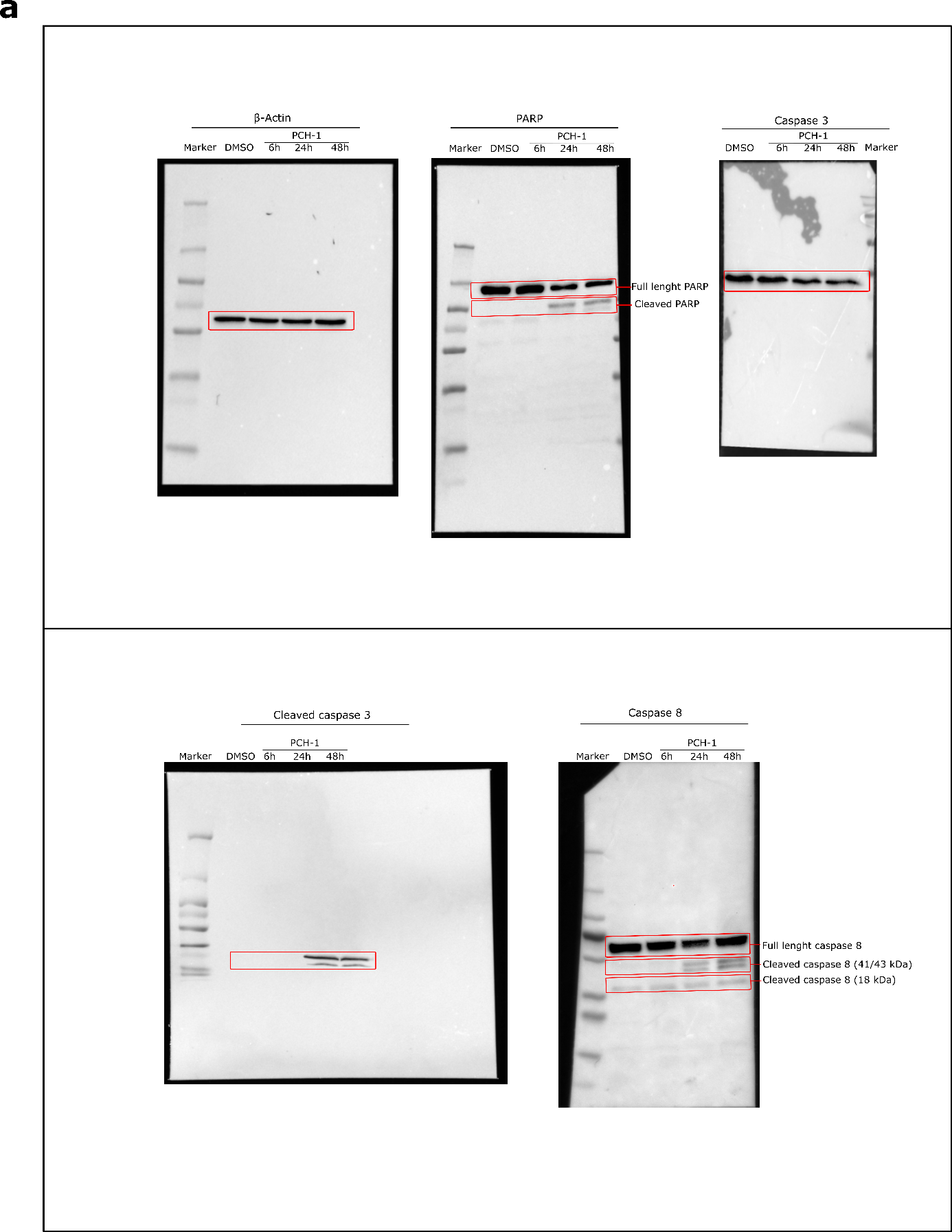


**Figure S4** **a**. Full length western blot presented in **Figure 6** of the main article. Red boxes denote the cropped regions of the blots.


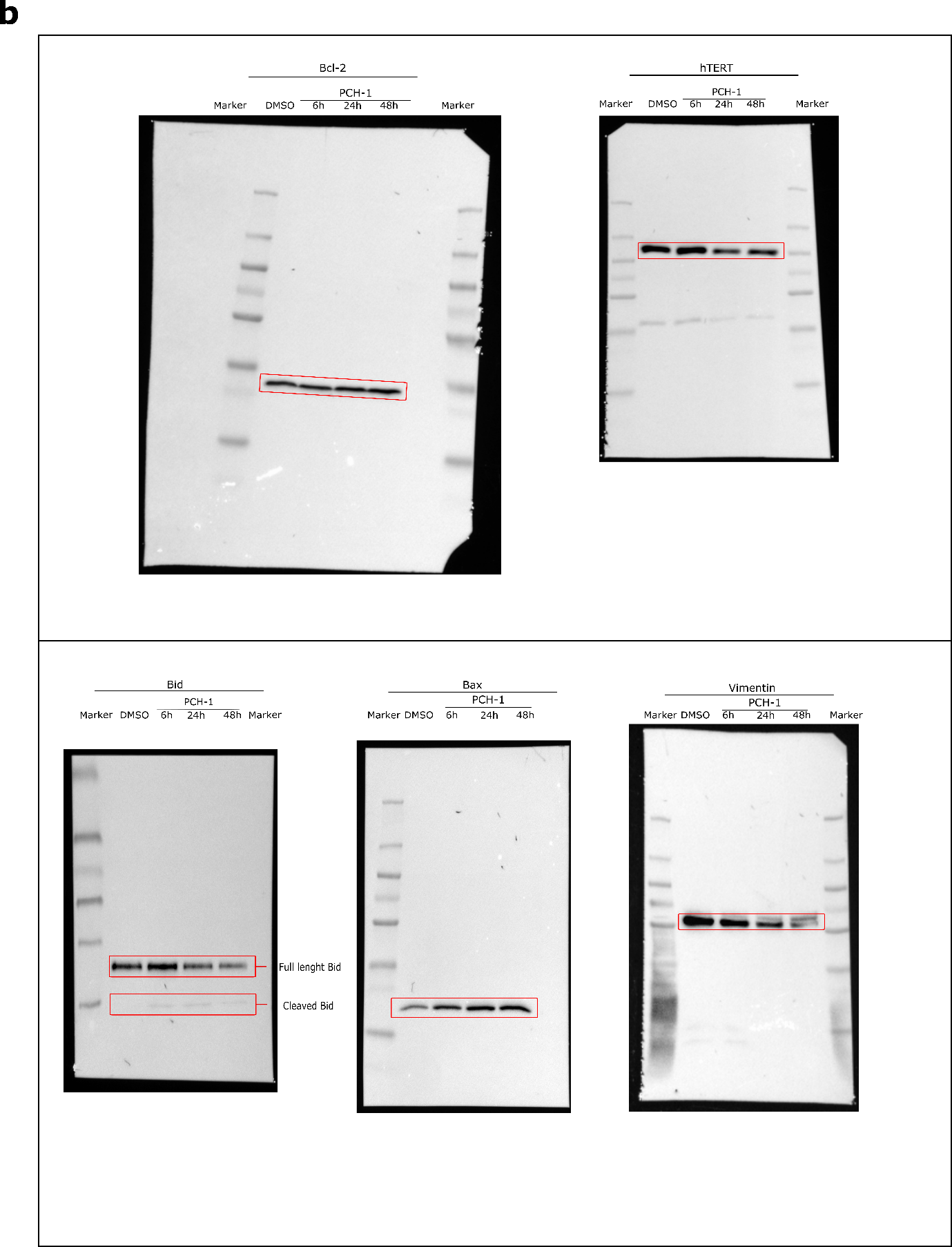


**Figure S4** **b**. Full length western blot presented in **Figure 6** of the main article. Red boxes denote the cropped regions of the blots.

*
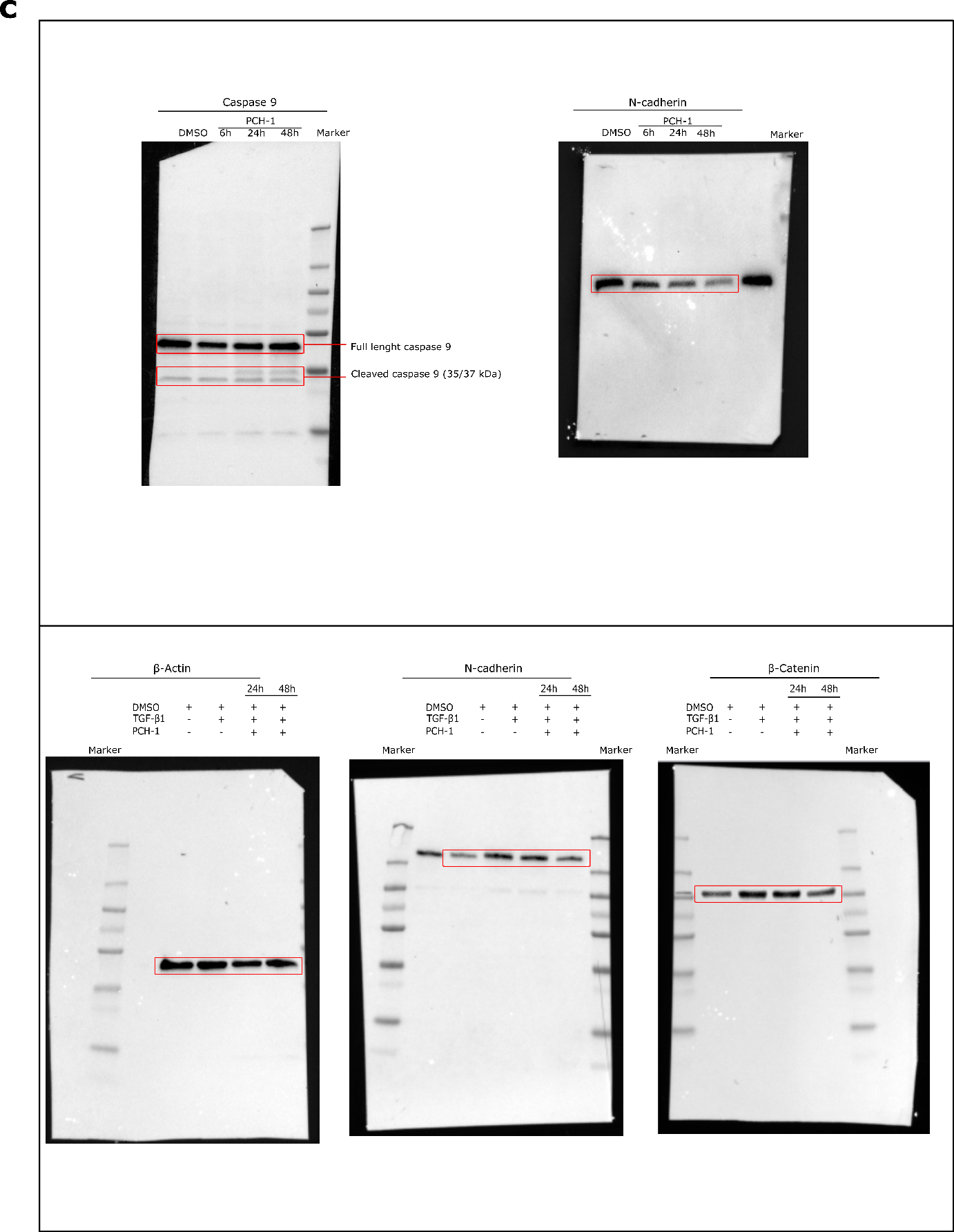
*

**Figure S4** **c**. Full length western blot presented in **Figure 6** of the main article. Red boxes denote the cropped regions of the blots.

*
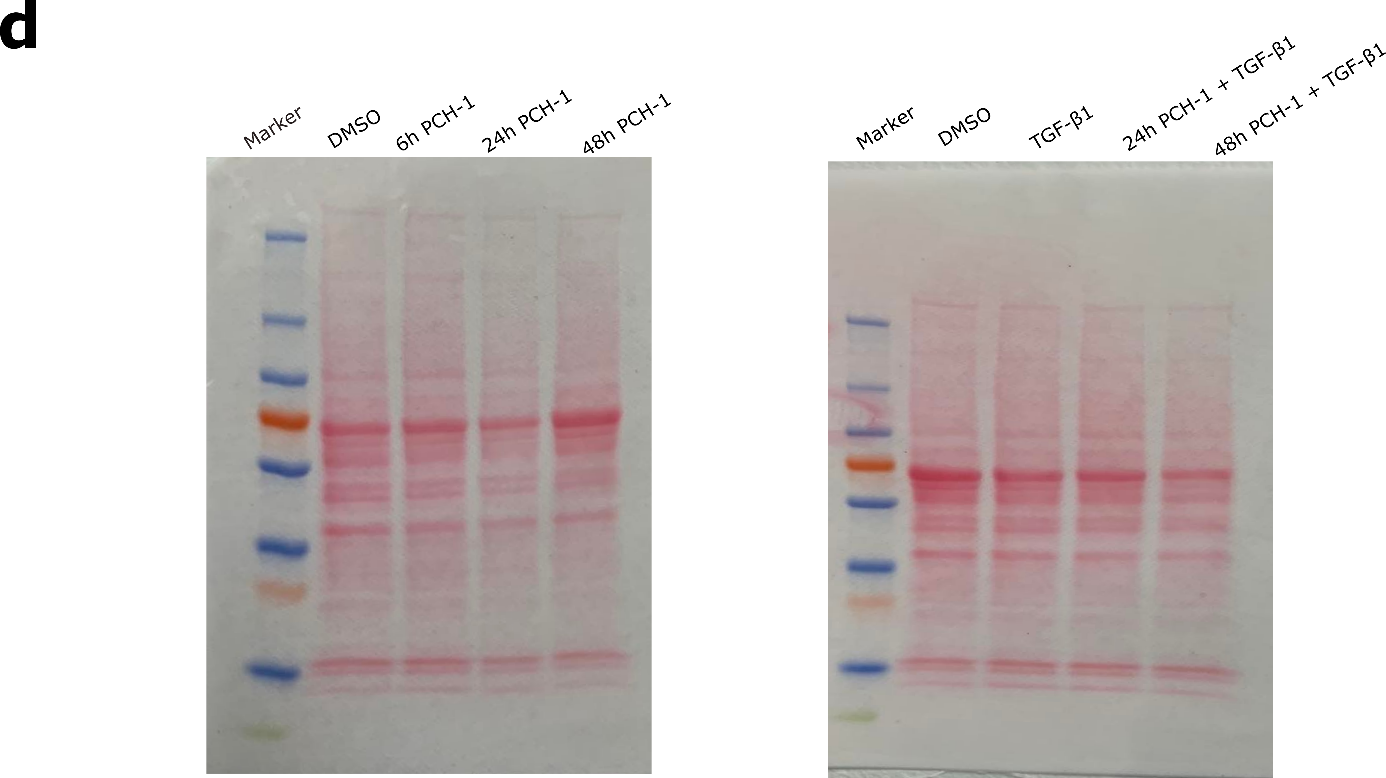
*

**Figure S4** **d**. Representative images of a nitrocellulose membranes stained with Ponceau S dye for protein detection during western blot.


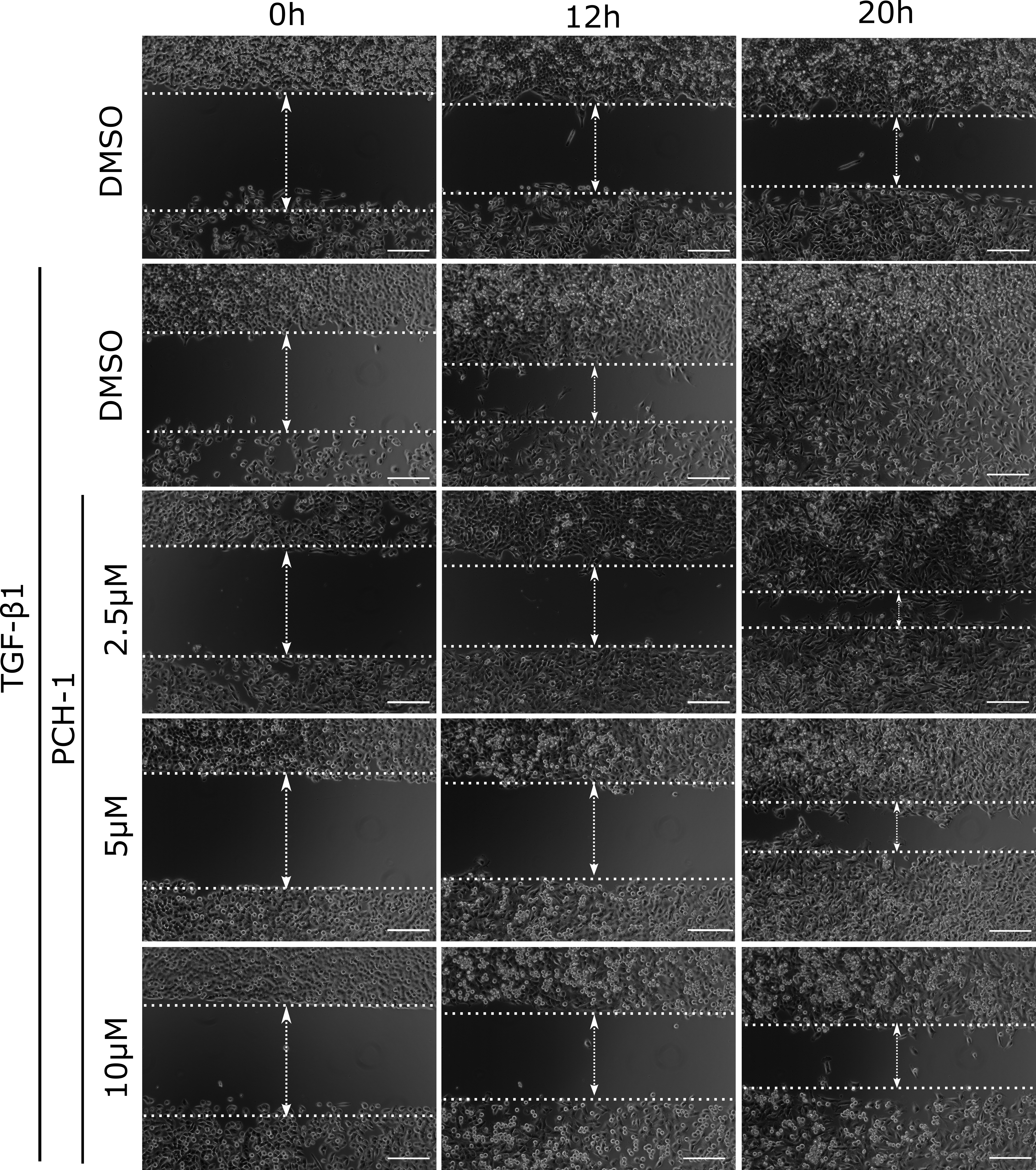


***Figure S5*** Analysis of TGF-β1 stimulated and unstimulated A-549 cell migration after treatment with PCH-1 by *in vitro* wound healing assay presented as time-lapse microscopy images after culture insert removal. Scale bars = 100 μm.

**
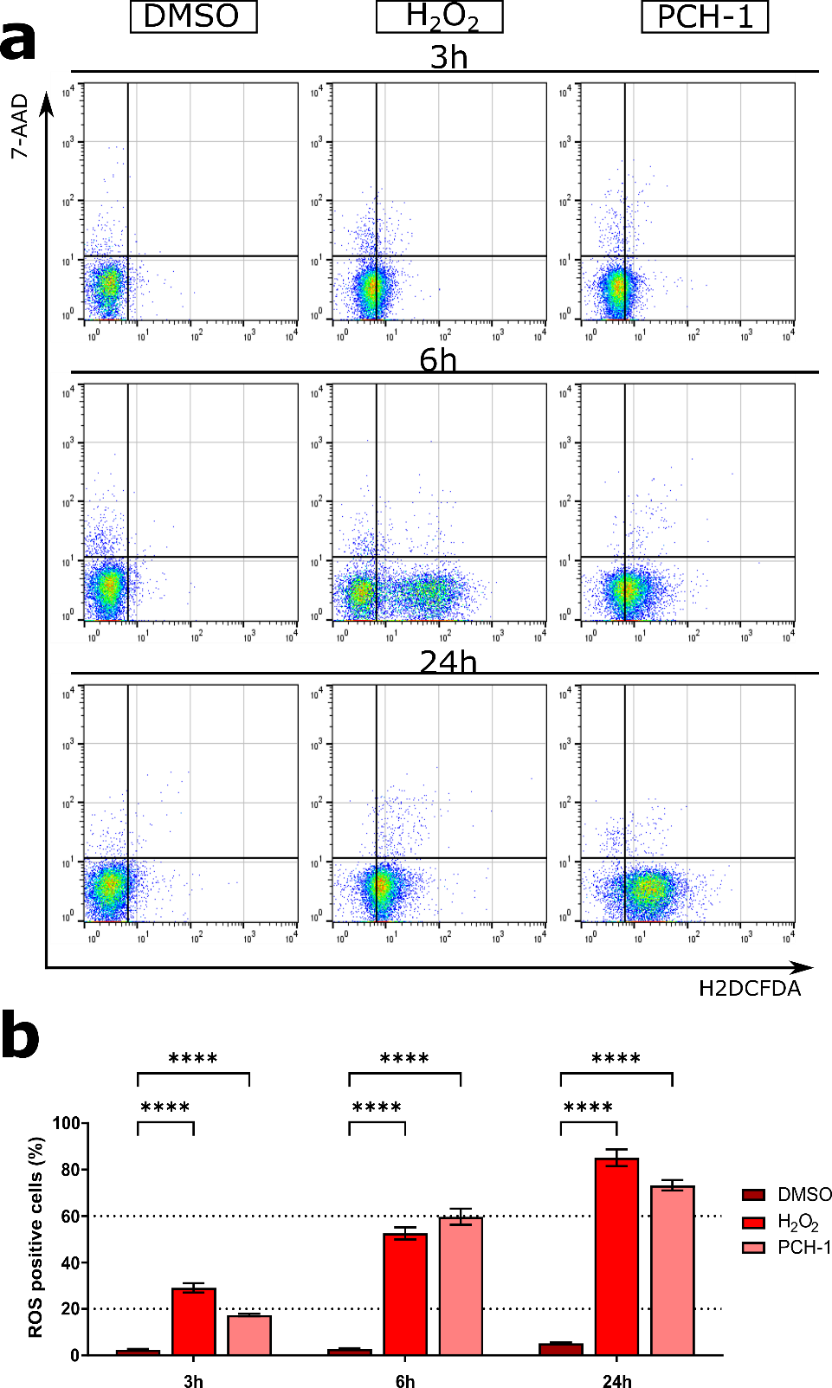
**

**Figure S6** Flow cytometry analyses of ROS level at 3, 6, and 24 h of treatment A-549 cells with **PCH-1**. **a**. Representative dot plots after labeling with H2DCFDA/7-AAD; **b**. The quantitation of the analysis is presented in a graph bar. Data represent the mean ± SEM of three independent experiments. Statistical differences were analyzed by one-way ANOVA and post hoc Dunnett`s test. ** p<0.001, *** p<0.0001, **** p< 0.00001.

**Methods**

**Table S1** List of antibodies used in Western Blot

| Antibody name | Company | Dilution |
| --- | --- | --- |
| Anti-Caspase-3 (#9662)  Anti-Cleavage caspase-3 (#9661) | Cell Signaling  Cell Signaling | 1:1000  1:1000 |
| Anti-Caspase-9 (#9502) | Cell Signaling | 1:1000 |
| Anti-Caspase-8 (1C12) (#9746) | Cell Signaling | 1:1000 |
| Anti-PARP (#9542) | Cell Signaling | 1:1000 |
| Anti-Bcl-2 (#4223) | Cell Signaling | 1:1000 |
| Anti-BID (#2002) | Cell Signaling | 1:1000 |
| Anti-Bax (#2772) | Cell Signaling | 1:1000 |
| Anti-Telomerase reverse  transcriptase antibody [Y182] (ab32020) | Cell Signaling | 1:1000 |
| Anti-Vimentin (#5741) | Cell Signaling | 1:1000 |
| Anti- β-Catenin (#9562) | Cell Signaling | 1:1000 |
| Anti-N-cadherin (610920) | BD Transduction Laboratories | 1:250 |
| Anti-actin (sc-1616) | Santa Cruz | 1:100 |
| Anti-mouse-HRP (715-035-150) | Jacson ImmunoResearch Labs | 1:10000 |
| Anti-rabbit-HRP (711-035-152) | Jacson ImmunoResearch Labs | 1:10000 |
| Anti-goat-HRP (705-036-147) | Jacson ImmunoResearch Labs | 1:10000 |
